# Platform trials in anaesthesia and perioperative medicine: a scoping review

**DOI:** 10.1016/j.bjao.2026.100569

**Published:** 2026-06-02

**Authors:** Pooveshni Govender, Daniel R. Frei, Hassan M. Ahmed, Richard Beasley, Elizabeth G. Ryan, Paul S. Myles

**Affiliations:** 1 Department of Anaesthesia and Pain Management, Wellington Regional Hospital, Wellington, New Zealand; 2 Department of Anaesthesia, Faculty of Medicine, Cairo University, Cairo, Egypt; 3 Department of Anaesthesia, Leeds Teaching Hospitals NHS Trust, Leeds, United Kingdom; 4 Medical Research Institute of New Zealand, Wellington, New Zealand; 5 School of Public Health and Preventive Medicine, Monash University, Melbourne, Victoria, Australia; 6 Department of Anaesthesiology and Peri-operative Medicine, Alfred Health, Melbourne, Victoria, Australia; 7 Department of Anaesthesiology and Peri-operative Medicine, Monash University, Melbourne, Victoria, Australia

**Keywords:** adaptive trial designs, anaesthesiology, master protocol, perioperative medicine, platform trials, surgery

## Abstract

**Background:**

Traditional randomized control trials underpin evidence generation in anaesthesia and perioperative medicine, but are often poorly suited to evaluating multiple, evolving clinical questions. Platform trials encorporate a trial design that evaluates multiple interventions within one or more domains, is governed centrally using a master or core protocol, and has the capacity to add new research questions through the addition of new treatments and/or subgroup. Platform trials offer a flexible alternative, enabling the concurrent and sequential evaluation of multiple interventions and more efficient identification of effective or harmful treatments.

**Methods:**

We conducted a scoping review using the Joanna Briggs Institute methodology, reported in accordance with PRISMA-ScR. Searches of bibliographic databases, grey literature and clinical trial registries (Ovid MEDLINE, Ovid Embase, Scopus, CINAHL Complete, Web of Science and the Cochrane Library) were undertaken. Records meeting an a priori definition of platform trials in anaesthesia or perioperative medicine were included. Trial design and methodological features were extracted and synthesised descriptively.

**Results:**

Among 32 062 records identified, nine platform trials met eligibility criteria. Most were registered (8/9) and university-sponsored, with nearly half in the planning phase. All trials were multicentre, predominantly conducted in high-income countries and 44% involved international collaboration. Adult surgical populations were most commonly studied, with surgical site infection prevention being the leading perioperative focus. Across the nine platform trials, 24 interventions were evaluated, predominantly surgical (58.3%), followed by anaesthetic (12.5%). Adaptive features were universal, and Bayesian statistical methods predominated.

**Conclusions:**

Platform trials remain uncommon and methodologically diverse in anaesthesia and perioperative medicine, reflecting early-stage adoption within an evolving field. Greater consensus regarding nomenclature, governance, and methodological standards, alongside expansion into anaesthesia-specific domains, is needed to realise their efficiency and ethical advantages.

Randomized controlled trials (RCTs) play a central role in medical innovation, the advancement of clinical care, and improvements in patient-centred outcomes.^1^, ^2^, ^3^ They are regarded as the most rigorous method for establishing causal relationships between interventions and outcomes, and provide the highest level of evidence for evaluating the effectiveness of new or existing treatments, strategies, and clinical practices.^1^, ^2^, ^3^ Despite their crucial role, they are frequently criticised for being time-consuming, costly, limited in the range of clinical questions they can address, and slow to translate findings into routine clinical practice.^2^^,^^4^ Innovative trial designs, particularly platform trials, have emerged as a potential solution to some of the limitations of traditional RCTs.^5^, ^6^, ^7^

Although no universal definition exists, platform trials are consistently characterised by the evaluation of multiple interventions within one or more domains (therapeutic area within which several interventions are compared, typically a drug or treatment class), often across different participant subgroups defined by strata (baseline patient-specific characteristics) and states (disease-specific characteristics).^5^^,^^7^, ^8^, ^9^, ^10^, ^11^ Platform trials are conducted under a centrally governed master or core protocol, with the in-built capacity to incorporate new research questions by adding new interventions and/or subgroups.^5^^,^^7^, ^8^, ^9^, ^10^, ^11^ The overarching design is intended to be perpetual and remain operational over time.^4^^,^^5^^,^^7^^,^^9^, ^10^, ^11^, ^12^

Many platform trials incorporate adaptive features, defined as prespecified modifications to elements of the trial design based on accumulating data, typically assessed at interim analyses (e.g. arm dropping, domain closure, response adaptive randomisation).^8^^,^^12^ However, these adaptive features are not a defining characteristic of platform trials but rather methodological features that may be incorporated within their structural framework.^8^^,^^12^ As a result, platform trials offer several advantages, including increased flexibility, more efficient evaluation of treatments, potentially reduced costs through the use of shared infrastructure, and a greater focus on patient-centred outcomes.^1^^,^^5^^,^^7^^,^^9^^,^^13^, ^14^, ^15^, ^16^, ^17^ These characteristics are useful for research in anaesthesia and perioperative medicine, where numerous unresolved clinical questions coexist across the perioperative continuum.^1^, ^2^, ^3^^,^^5^^,^^13^^,^^14^^,^^16^^,^^18^, ^19^, ^20^, ^21^

This scoping review aims to establish a speciality-specific baseline by identifying existing platform trials in anaesthesia and perioperative medicine, characterising their design features, and identifying methodological and clinical gaps to highlight opportunities for future anaesthesia-led platform trials.

## Methods

This scoping review was reported according to the Preferred Reporting Items for Systematic Reviews and Meta-Analyses guidelines extension for scoping reviews (PRISMA-ScR) (Supplementary Fig. 1).^22^ The protocol was developed using the Joanna Briggs Institute (JBI) methodology for scoping reviews, finalised and registered on Open Science Framework (OSF, 27 February 2025) before any formal screening or data extraction commenced.^23^^,^^24^ Where searches were updated after registration, this has been explicitly stated. Research ethics board approval was not required as no patient-level data were accessed.

### Search strategies

An initial literature search was undertaken in Ovid MEDLINE on 6 November 2024 to identify terminology and inform keyword development. These terms were refined and incorporated into the final comprehensive search strategy on 12 February 2025 with support from a research librarian. The strategy combined free-text terms with Boolean operators and was intentionally broad, reflecting the lack of standardised nomenclature for platform trials in the current literature. Critical care terminology was included in the search strategy to improve sensitivity where overlap with perioperative medicine was plausible. The search strategy was first run on 3 March 2025 and subsequently rerun on 10 November 2025. The dataset was locked immediately thereafter, and no additional records were included.

The search strategy was applied across Ovid MEDLINE, Ovid Embase, Scopus, CINAHL Complete, Web of Science, the Cochrane Library, and several clinical trial registries, including ClinicalTrials.gov, the International Standardised Randomised Controlled Trial Number Registry (ISRCTN), the Australian New Zealand Clinical Trials Registry (ANZCTR), the World Health Organization International Clinical Trials Registry Platform (ICTRP), and the National Institute for Health and Care Research (NIHR). To increase sensitivity, the websites of professional anaesthesia societies, academic institutions, government health agencies, grey literature servers (OSF, Zenodo and medRxiv), preprint servers (Europe PMC) and the reference lists of key articles were searched using predefined lists and keywords. The detailed search strategy is provided in Supplementary Fig. 2.

### Eligibility criteria

Records were eligible if they met an a priori definition of a platform trial in anaesthesia or perioperative medicine.

Perioperative medicine was defined as care delivered within a defined surgical episode, spanning the preoperative, intraoperative, and postoperative phases, and involving anaesthesia within a coordinated multidisciplinary pathway.^25^ Eligibility required that platform trials be explicitly designed to evaluate interventions targeting perioperative processes or outcomes within this surgical pathway. Trials were not considered perioperative if they primarily addressed broader populations (e.g. critical care), even where surgical patients were included, unless a clearly specified perioperative framework was integral to the trial design. Postoperative outcomes such as surgical site infection were considered eligible when they were directly addressed through perioperative interventions or trial pathways. Inclusion was therefore determined by trial design intent rather than patient population alone.

A platform trial was defined as a trial design that evaluates multiple interventions within one or more domains, is governed centrally using a master or core protocol, and has the capacity to add new research questions through the addition of new treatments and/or subgroups.^8^^,^^17^ Eligibility was assessed using the study protocol, trial registry entry, or published results. A prespecified decision framework was applied consistently across all records to ensure that included trials met both the structural definition of a platform trial and the requirement for explicit targeting of a perioperative surgical pathway (Supplementary Table 1).

Trials were not classified as platform trials if they: 1) were multi-arm adaptive RCTs without a master protocol; 2) did not have the capacity to address new research questions through the addition of new treatments and/or subgroups; or 3) incorporated umbrella or basket trial design elements and did not meet all prespecified defining criteria for a platform trial. Pain-related and critical care platform trials were eligible only if conducted within a clearly defined surgical perioperative pathway.

Disagreements were resolved through discussion and, where required, consultation with another reviewer (DF). These trials were excluded and documented in the screening log. No restrictions were imposed regarding publication date or language. Expert commentaries, perspective pieces, conference abstracts, and newsletters were excluded unless they were linked to an eligible registered platform trial. Any record not fulfilling all eligibility criteria was omitted.

### Selection of records

All retrieved records were imported into Covidence (Veritas Health Innovation, Melbourne, Australia), where duplicate records were removed.

Title and abstract screening, along with screening of trial registry entries, was performed in duplicate by three independent reviewers (PG, HA, and PB). Any disagreements were resolved through discussion or, when necessary, by consensus to determine final inclusion. Citation tracking, grey literature searches, and screening of preprint servers were conducted by a single reviewer (PG). When multiple records corresponded to a single platform trial, these were grouped under the trial’s acronym and official title. Once a platform trial was identified for inclusion, a targeted Google search was undertaken to determine whether an official trial website existed and to locate the master protocol if it had not been identified during the initial search.

### Data extraction

Data extraction was conducted by two reviewers (PG and HA), and any disagreements were resolved by consensus with a third reviewer (DF). A standardised data extraction tool was developed a priori, piloted on the first three records, and refined before full data extraction commenced (Supplementary Table 2). Extracted variables included trial characteristics, key design and methodological features, statistical and analytical approaches, and operational characteristics. Relevant data were extracted using Microsoft Excel (v16, Microsoft Corp., Redmond, WA, USA). Where platform trials had multiple associated records (e.g. protocol, registry entry, primary report, differing versions of protocols), data were reconciled under the trial’s acronym and official title, with the most recent version prioritised.

Data extraction was restricted to publicly accessible sources, including trial registries, published protocols, peer-reviewed publications, trial websites, and relevant grey literature. Investigators were not contacted for additional information, as the objective of this scoping review was to characterise the platform trial landscape based on publicly available data. This approach was chosen to ensure methodological consistency and reproducibility, and to minimise the potential for selection or response bias arising from differential investigator engagement.

### Data analysis

Microsoft Excel (v16, Microsoft Corp., Redmond, WA, USA) was used to synthesise the data. All included platform trials were summarised narratively in tabulated format. The geographic distribution of registered platform trials is illustrated using a world choropleth map. For each included platform trial, countries were coded according to locations where participant recruitment occurred. Trials recruiting across multiple countries contributed one count to each participating country. The outcomes are displayed to represent the absolute number of platform trials per country (not population-adjusted). Bar graphs are used to illustrate the number of identified trials stratified by trial status (planning, recruiting, terminated or completed) and 1) primary perioperative focus and 2) surgical speciality.

Descriptive data are presented as a number (proportion) or median (inter-quartile range). The heterogeneity of platform trials in anaesthesia and perioperative medicine precluded quantitative synthesis; hence, this scoping review did not assess comparative effectiveness or risk of bias. Data analysis was conducted using IBM SPSS Statistics, version 31.0 (IBM Corp., Armonk, NY).

## Results

**Fig 1:**
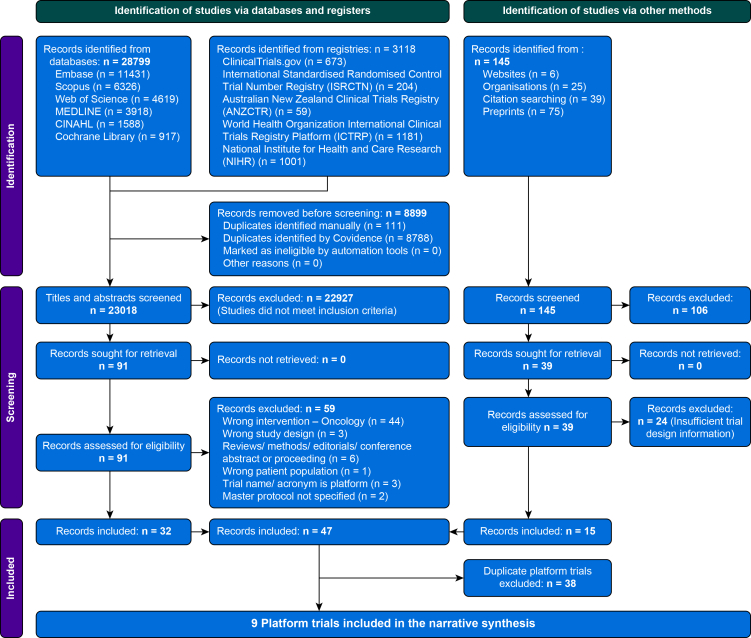
PRISMA flowchart demonstrating the full scoping review process from initial search to abstract screening, full text assessment and grey literature screening. PRISMA, Preferred Reporting Items for Systematic Reviews and Meta-Analyses.

The final comprehensive search strategy yielded 32 062 records (Fig. 1). After removal of duplicates, 23 018 records were screened by title and abstract, of which 22 927 were excluded. A total of 130 records (91 from database/register sources and 39 from grey literature sources) underwent full-text eligibility assessment. Reasons for exclusion are summarised in the PRISMA flow diagram (Fig. 1). Overall, 47 records were included in the analysis. Where multiple records related to a single platform trial, these were consolidated under the trial’s acronym and official title. This process resulted in the inclusion of nine platform trials in the final narrative synthesis. (Table 1 and Supplementary Table 3).

**Table 1:** Narrative summary of platform trials in anaesthesia and perioperative medicine. AEGIS, analgesia with ibuprofen for patients undergoing elective major gastrointestinal surgery; HFNO, high flow nasal oxygen for patients undergoing elective major abdominal surgery; MAMS, multiarm, multistage; PONV, postoperative nausea and vomiting; REMAP, randomised, embedded, multifactorial, adaptive platform; SPRY, strategies to promote resiliency.∗Full trial titles provided in Supplementary Table 3. ^†^Primary perioperative focus reflects the overarching clinical problem targeted by the platform.

Platform trial (acronym)^∗^	Registration	Trial status	Trial site/s	Population	Surgical speciality	Primary perioperative focus^†^	Perioperative domains	Trial design framework
UPMC REMAP^26^	NCT03861767; STUDY19090186	Terminated	Multicentre national	Adults	Mixed	Perioperative therapies	SPRY -Metformin	REMAP
MARLIN^27^	NCT06465901; RG_23-086	Planning	Multicentre international	Adults and children (≥ 5 yr)	Abdominal	Surgical site infection		Pragmatic adaptive platform RCT; MAMS; 2x2x2 factorial; outcome assessor blinded
PROMPT^28^	NCT07186634; 2025-49324-130781	Planning	Multicentre international	Adults	Mixed	Surgical site infection	Oxygen	Pragmatic adaptive platform RCT; MAMS; multifactorial
PROTECT-Surg^29^	NCT04386070; RG_20-029 COVID-19; 2020-001448-24	Terminated	Multicentre international	Adults	Abdominal; Thoracic	Postoperative pulmonary complications		Pragmatic adaptive platform RCT
REMAP-Periop^30^	NCT04606264; STUDY19030022	Completed	Multicentre national	Adults	Abdominal	Enhanced recovery pathways	PONV prophylaxis; Regional/ neuraxial analgesia	REMAP
ROADMAP^31^	NCT06771050; R-2024-0041	Planning	Multicentre international	Adults	Orthopaedic	Surgical site infection	Surgical; Antibiotic choice; Antibiotic duration	REMAP
ROSSINI 2^32^	NCT03838575; ISRCTN78305317; IRAS247285; CPMS39722	Recruiting	Multicentre national	Adults	Abdominal	Surgical site infection	Abdominal surgery	Pragmatic adaptive platform RCT; MAMS design
ROSSINI-Platform^33^		Planning	Multicentre national	Adults	Mixed	Surgical site infection	Vascular groin; Lower limb amputation; Obstetric; Breast; Neurosurgery; Cardiac surgery	Adaptive platform RCT; basket MAMS design; multifactorial
PROTECT^34^	ISRCTN14639555; IRAS353122	Recruiting	Multicentre national	Adults	Mixed	Perioperative outcomes	AEGIS; HFNO; Diversity	Pragmatic adaptive platform RCT

The majority (8/9, 88.9%) of platform trials in anaesthesia and perioperative medicine were registered at the time of data extraction, and all were sponsored by university-affiliated institutions (Table 2). Trial status varied, with four trials (44.4%) in the planning phase, two (22.2%) recruiting, two (22.2%) terminated and one (11.1%) completed. Five platform trials (55.6%) had a publicly accessible trial website with an available master or core protocol.

**Table 2:** Summary of the characteristics of platform trials in anaesthesia and perioperative medicine. Data are presented as *n* (%) or median (IQR [range]). ∗Type of interventions across platform trials, where other includes perioperative drugs (*n*=6) and consent process (*n*=1). eCRF, electronic case report form; IQR, inter-quartile range; HIC, high-income country; LMIC, lower-middle-income country; UMIC, upper-middle-income country.

Trial characteristic	Description	*n* (%)
Master protocol	Publicly available	5 (55.6)
Trial registration		8 (88.9)
Population	Adults	8 (88.9)
	Adults + children	1 (11.1)
Planned sample size for platform trial	≤ 999	1 (11.1)
	1000–9999	6 (66.7)
	≥10 000	2 (22.2)
Number of domains per platform trial	Single	2 (22.2)
	Multiple	7 (77.8)
Starting number of platform trial arms		6 (3.8 – 6.3 [2 – 11])
Total number of interventions across platform trials		24
Type of interventions across platform trials^∗^	Anaesthetic	3 (12.5)
	Surgical	14 (58.3)
	Other	7 (29.2)
Number of platform trials with strata		4 (44.4)
Statistical model	Bayesian	5 (55.6)
	Frequentist	2 (22.2)
	Both	1 (11.1)
	Not reported	1 (11.1)
Adaptive features used	Yes	9 (100)
Type of adaptive features	Response adaptive randomisation	5 (55.6)
	Prespecified adaptation rules	6 (66.7)
	Arm dropping	7 (77.8)
	Enrichment	1 (11.1)
	Adding new interventions	6 (66.7)
	Sample size re-estimation	2 (22.2)
	Perpetual design	5 (55.6)
	Prespecified interim analysis	6 (66.7)
	Trial adjustments made at interim analysis	3 (33.3)
Trial status	Planning	4 (44.4)
	Recruiting	2 (22.2)
	Terminated	2 (22.2)
	Completed	1 (11.1)
Study sponsor	University affiliation	9 (100)
Geographic setting	HIC	7 (77.8)
	LMIC	2 (22.2)
Trial sites	Multicentre + national	5 (55.6)
	Multicentre + international	4 (44.4)
Governance and operations committee	Yes	9 (100)
Reporting standards documented		6 (66.7)
Database	Embedding	2 (22.2)
	Central database	3 (33.3)
	Not reported	4 (44.4)
Published results available		3 (33.3)
Trial website available		5 (55.6)
Funding source	Academic	2 (22.2)
	Government	4 (44.4)
	Both	1 (11.1)
	Not reported	2 (22.2)

**Fig 2:**
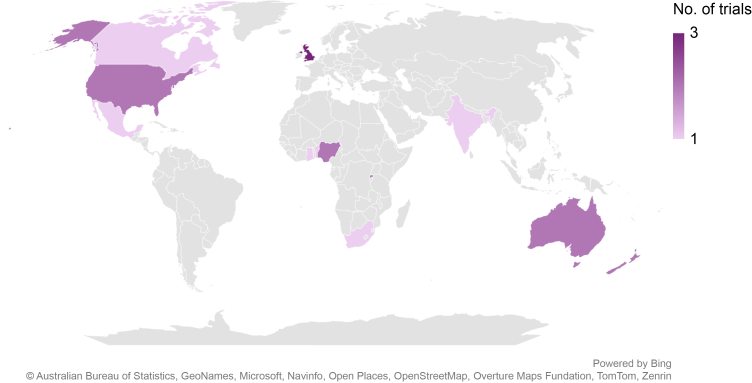
Geographic distribution of platform trials in anaesthesia and perioperative medicine. Countries are shaded according to the number of anaesthesia and perioperative platform trials with recruiting sites in each country. Trials recruiting across multiple countries contributed one count to each participating country. The outcome displayed represents the absolute number of platform trials per country (not population-adjusted). Geographic distribution may differ from sponsor or coordinating centre location.

The geographic distribution of platform trials being undertaken worldwide was broad, with most activity in North America, the United Kingdom and Australasia (Fig. 2). All platform trials were multicentre, with four trials (44.4%) involving international collaborative networks (Table 2). Most trials were concentrated in high-income countries (7/9, 77.8%) with decreased representation from lower-middle-income countries (2/9, 22.2%). Trials were categorised according to the income level of participating countries using the World Bank country income classification at the time of data extraction (Supplementary Table 4).

**Fig 3:**
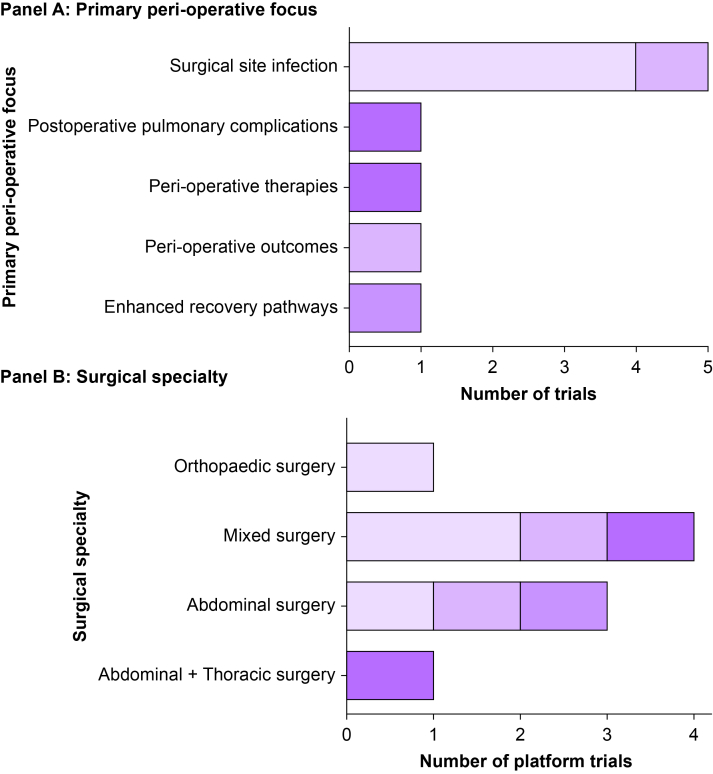
Distribution of perioperative platform trials by primary perioperative focus and surgical specialty. Bars represent the number of perioperative platform trials and are shaded according to trial status, shown using progressively darker shades of purple from planning (light purple), recruiting, completed to terminated trials (dark purple). Panel A shows trials categorised by primary perioperative focus, and Panel B shows trials categorised by surgical specialty.

Eight (88.9%) platform trials were conducted in adult patient populations across a range of surgical specialities: abdominal (3/9, 33.3%), orthopaedic (1/9, 11.1%), combined abdominal and/or thoracic (1/9, 11.1%) and mixed (4/9, 44.4%) (Table 1). Surgical site infection (SSI) prevention emerged as the predominant primary perioperative focus, accounting for 55.6% (5/9) of all included platform trials, whilst fewer platform trials were focused on postoperative pulmonary complications (1/9, 11.1%), perioperative therapies (1/9, 11.1%), perioperative outcomes (1/9, 11.1%) and enhanced recovery pathways (1/9, 11.1%) (Fig. 3).

Planned trial sample sizes ranged from 1 000 to 9 999 participants in 66.7% (6/9) of included platform trials, whilst 22.2% (2/9) were designed for a sample size of ≥10 000 participants (Table 2). Most trials (7/9, 77.8%) incorporated multiple domains into the trial infrastructure, with single-domain platform trials accounting for 22.2% (2/9). The starting number of arms varied across included platform trials, with a median of 6 (3.8 – 6.3 [2 – 11]). There were 24 documented interventions across all platform trials, including anaesthetic (3/24, 12.5%), surgical (14/24, 58.3%) and other interventions (7/24, 29.2%) such as perioperative drug administration and use of multi-lingual consent forms. Published results were available from three platform trials (33.3%).

Bayesian methods (5/9, 55.6%) were the dominant statistical model incorporated into the platform trial design compared with frequentist statistical methods (2/9, 22.2%) (Table 2). Five platform trials (55.6%) reported a perpetual design. Six trials (66.7%) documented details for prespecified interim analyses, and three (33.3%) reported trial adjustments made at one or more interim analyses. Adaptive features were integrated into all included platform trials. These features included: response adaptive randomisation (5/9, 55.6%), prespecified adaptation rules (6/9, 66.7%), arm dropping (7/9, 77.8%), enrichment (1/9, 11.1%), adding new interventions (6/9, 66.7%) and sample size re-estimation (2/9, 22.2%).

All platform trials reported the presence of governance and operations committees, but only six (66.7%) documented use of reporting standards (Table 2). Data infrastructure approaches varied, including the use of embedded data systems (2/9, 22.2%) and centralised database structures (3/9, 33.3%), while four trials (44.4%) did not report data infrastructure details (Supplementary Table 5 and 6). Most platform trials received government funding (4/9, 44.4%) followed by academic sources of funding (2/9, 22.2%).

## Discussion

Despite increasing interest, platform trials remain rare, heterogeneous, and at an early stage of development in anaesthesia and perioperative medicine. In this scoping review, nine platform trials were identified, highlighting inconsistency in nomenclature, limited population and geographic diversity, and marked variation in methodological maturity and governance.^26^, ^27^, ^28^, ^29^, ^30^, ^31^, ^32^, ^33^, ^34^

These findings reflect the early stage of platform trial adoption within anaesthesia and perioperative medicine. Platform trials have been conducted more commonly in fields such as oncology and infectious diseases (COVID-19); however, speciality-adjusted comparisons are not currently available.^9^ Accordingly, platform trial methodology remains at an early stage of development within anaesthesia and perioperative medicine, with clear opportunities for future development.

**Fig 4:**
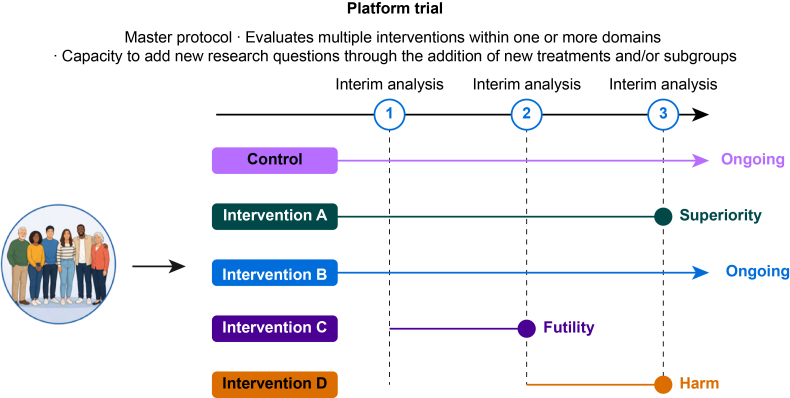
Schematic representation of a single-domain platform trial comparing two interventions (A and B) with a shared control group. The control group may comprise all control participants accrued over the course of the trial (including both concurrent and non-concurrent controls) or be restricted to those enrolled and randomised within the same time period as the intervention under evaluation (concurrent controls). Interventions may enter or exit the platform at different time points based on accumulating evidence. Adaptive methodological features, including prespecified stopping rules, are applied to specific interventions (A, C, and D), such as efficacy (where an intervention is superior to control), futility (where an intervention is ineffective or unlikely to demonstrate benefit), and harm (where an intervention is associated with adverse outcomes), resulting in changes to the set of active interventions over time. The platform trial is designed to be perpetual.

This early-stage development likely reflects several underlying methodological and conceptual challenges, of which persistent definitional ambiguity is a contributor. Inconsistent use of terms such as “platform,” “multi-arm,” and “adaptive” complicates classification and risks conflating platform trials with other adaptive trial designs.^9^^,^^12^^,^^35^, ^36^, ^37^, ^38^ Many platform trials incorporate adaptive features, defined as prespecified modifications to elements of the trial design based on accumulating data, typically assessed at interim analyses (e.g. arm dropping, domain closure, response-adaptive randomisation).^8^^,^^12^ However, these adaptive features represent methodological components rather than defining characteristics of platform trials.^7^, ^8^, ^9^, ^10^, ^11^, ^12^^,^^17^ Platform trials are fundamentally defined by their structural framework, including a centrally governed master protocol, evaluation of multiple interventions within one or more domains, and the capacity to incorporate new research questions over time by adding new interventions and/or subgroups (Fig. 4).^7^, ^8^, ^9^, ^10^, ^11^, ^12^ While all included trials incorporated adaptive features, this likely reflects current practice rather than a necessary requirement of platform trial design, reinforcing the importance of distinguishing between structure and methodology when defining these trials.^17^ To address this, we applied a decision framework to identify true platform trials.^5^^,^^7^, ^8^, ^9^^,^^11^ Nevertheless, variability in reporting and incomplete public documentation limited transparency in several cases. Establishing consensus terminology and minimum defining criteria, aligned with emerging methodological guidance, will be essential to improve reproducibility, facilitate cross-trial comparison, and support regulatory and ethical oversight.^7^, ^8^, ^9^^,^^12^^,^^17^

Beyond definitional challenges, important gaps in population representation persist across anaesthesia and perioperative platform trial research.^1^^,^^21^ This study found that physiologically distinct or high-risk populations, such as patients undergoing cardiac and thoracic surgery, obstetric populations, older adults with frailty or multimorbidity, and paediatric cohorts, remain under-studied. These groups exhibit distinct physiological responses to anaesthesia and surgery and experience a disproportionate burden of perioperative morbidity.^2^^,^^3^^,^^21^^,^^39^, ^40^, ^41^, ^42^, ^43^, ^44^ Emerging platform trials, such as the Paediatric Adaptive Sepsis Platform Trial (PASSPORT) and the Paediatric Intensive Care Adaptive Platform Trial (PIVOTAL), illustrate the value of innovative trial designs when applied to physiologically distinct populations.^44^, ^45^, ^46^ However, comparable applications within anaesthesia and perioperative research remain limited.^1^^,^^20^^,^^21^

Contemporary platform trials such as the ROSSINI-Platform may address some of these gaps, although their impact remains uncertain given the early stage of development.^33^ Consequently, the limited representation of anaesthesia-specific platform trials in physiologically distinct or high-risk populations represents a persistent evidence gap and a clear opportunity for anaesthesia-led research groups to shape the future application of platform methodologies within the specialty.^1^, ^2^, ^3^^,^^20^^,^^21^^,^^39^, ^40^, ^41^, ^42^, ^43^

Similarly, the geographic distribution of platform trials warrants critical reflection. Platform trials are often promoted as efficient and generalisable, yet current evidence does not reflect global perioperative practice.^20^ Most platform trials are being undertaken in high-income countries with comparatively limited representation from low- and middle-income countries. As a result, much of the practical experience and methodological expertise in the design and conduct of platform trials likely resides within investigators and institutions based in high-income countries.^47^ Future platform trials should explicitly incorporate strategies for equitable collaboration, including shared leadership, capacity building, and context-specific adaptation, to ensure that efficiency gains do not exacerbate existing research inequities.^47^

The clinical focus of existing platform trials further illustrates both promise and imbalance. Surgical site infection (SSI) prevention was the most common primary perioperative focus identified. SSIs account for a substantial proportion of perioperative morbidity, mortality, and health care resource utilisation.^48^, ^49^, ^50^ Given the importance of this research focus, platform trials represent a particularly useful method for answering multiple related questions, owing to their efficiency, adaptability, and capacity to test several interventions concurrently.^1^ The range of surgical specialities represented further reinforces the suitability of platform designs for multidisciplinary application and enhances the potential external validity of trial findings.^9^^,^^51^ Although the predominance of SSI-focused platform trials reflects a significant clinical burden, numerous other potentially beneficial interventions remain underexplored. Anaesthesia-specific domains, including analgesia and pain-related outcomes, metabolic optimisation, and perioperative organ protection, are equally well-suited to adaptive, multi-arm evaluation yet remain underrepresented.^1^ This imbalance highlights missed opportunities to apply platform trial designs to other high-yield perioperative research questions, but also represents a promising area for future development, given that this trial design remains in the early stages of adoption in this field.^1^

From an operational perspective, all included trials were multicentre and sponsored by university-affiliated institutions, highlighting the reliance on established academic infrastructure. At the time of data extraction, only a small proportion were actively recruiting or completed, with some trials having been terminated. Reasons for trial termination included challenges with participant recruitment during the COVID-19 pandemic and difficulties in institutional medicine procurement.^26^^,^^29^ Hence, despite clear ethical advantages, including reduced exposure to inferior treatments, more efficient use of participant contributions through shared control groups, and adaptability to emerging evidence, platform trials can be operationally complex and resource-intensive.^1^^,^^12^^,^^20^^,^^52^ They may require substantial data management, administrative coordination, and advanced statistical expertise, and could frequently face funding and governance hurdles.^9^^,^^52^^,^^53^ Additional challenges include potential bias arising from time-varying control groups or non-concurrent intervention, prolonged durations that risk site and investigator fatigue, and variable acceptance with regulators and industry partners.^15^^,^^52^ Therefore, platform trials have the potential to be resource-intensive to design, implement, and sustain despite their many benefits.^6^^,^^19^

Consistent with this complexity, the extent and nature of adaptive features varied widely across trials. Interim analyses and arm dropping were commonly prespecified, whereas sample size re-estimation and enrichment strategies were less frequently reported, likely reflecting the early-stage nature of many platform trials.^5^^,^^10^^,^^11^ Bayesian statistical approaches predominated, consistent with the flexibility required for adaptive decision-making, but some trials employed frequentist methods.^5^^,^^10^^,^^11^

Governance and reporting practices were also highly variable. Platform trials are governed through multi-layered structures comprising strategic oversight, independent monitoring, operational management, and central trial coordination.^4^^,^^54^ Despite variation in terminology (e.g. trial management group, trial steering committee, data monitoring committee, data monitoring and ethics board, data and safety monitoring board), these components fulfil consistent core functions.^4^^,^^54^ However, limited protocol transparency, heterogeneity in reporting practices and varied data infrastructure approaches were noted, with some trials providing no publicly available information on data management. Collectively, these findings suggest that while methodological innovation is advancing in platform trial design, transparency and standardisation in governance, reporting, and data management are a work in progress.^4^^,^^51^^,^^54^^,^^55^ Improving transparency, standardised reporting and shared data platforms has the potential to strengthen patient safety oversight, enhance regulatory confidence, and support wider adoption of platform trials within anaesthesia and perioperative medicine.^4^^,^^51^^,^^54^^,^^55^

This scoping review has several strengths. It provides the first comprehensive overview of platform trials in anaesthesia and perioperative medicine and is supported by a rigorous and inclusive search strategy. However, several limitations warrant discussion. Consistent with the objectives of a scoping review, this work focused on mapping design characteristics rather than evaluating comparative effectiveness or risk of bias and findings should not be interpreted as comparative evaluations of trial quality or outcomes.

The search strategy was intentionally broad to maximise sensitivity, incorporating critical care terminology where overlap with perioperative medicine was plausible. Eligibility required a clearly defined perioperative surgical pathway, not just the presence of surgical patients within a broader critical care population. This definition prioritised trials explicitly designed around a surgical perioperative pathway, recognising that the inclusion of surgical patients alone does not constitute perioperative trial design. Consequently, several well-known adaptive platform trials identified during screening were excluded because they primarily involved critical care populations without a clearly defined surgical perioperative pathway, e.g. REMAP-CAP (Randomized Embedded Multifactorial Adaptive Platform trial for Community Acquired Pneumonia), INCEPT (Intensive Care Platform Trial), EMPRESS (Empirical Meropenem *vs* Piperacillin/Tazobactam for Adult Patients with Sepsis) and Paediatric Intensive Care Adaptive Platform Trial (PIVOTAL).^16^^,^^46^ In addition, some trials within an anaesthesia and a surgical perioperative pathway were not included as platform trials due to the absence of an explicitly described master protocol in publicly available sources, e.g. Anaesthesia and Perioperative Neurocognitive Disorders in the Elderly Patients Undergoing Hip Fracture Surgery Platform Trial (ANDES Platform Trial) and A Phase II Platform Trial of Perioperative Therapies in Locally Advanced Unresectable Gastric Cancer (Neo-VIKTORY).^56^^,^^57^

This review was limited to publicly accessible data sources, which varied in completeness. While additional operational details might have been obtained through direct contact with investigators, restricting the analysis to public sources ensured reproducibility and minimised potential selection and response bias. Consequently, some operational characteristics remain incompletely described and represent important avenues for future research.

Finally, additional platform trials may have been initiated after the final search, and existing platform trials continue to evolve over time. Consolidation of multiple records for the same platform trial may have obscured temporal changes, and the early stage of many platform trials limited assessment of feasibility, sustainability, and real-world impact.

## Conclusion

Platform trials represent a powerful methodological innovation with particular relevance to anaesthesia and perioperative medicine, yet their adoption across the speciality remains limited and uneven. This scoping review demonstrates that, despite growing interest, platform trials in this field are rare, methodologically heterogeneous, and largely confined to an early stage of development.

Realising the promised efficiencies and ethical advantages of platform trial designs will require deliberate, field-specific action. Priorities include establishing consensus nomenclature and minimum methodological standards, strengthening transparency in governance and reporting, expanding the inclusion of diverse populations and low- and middle-income settings, and extending platform methodology to anaesthesia-specific research domains where unmet clinical need remains substantial. Without coordinated investment and leadership, platform trials risk remaining isolated innovations rather than becoming embedded infrastructure for perioperative evidence generation. Addressing these challenges is therefore essential if anaesthesia and perioperative medicine are to harness platform trials as a transformative approach to efficient, equitable, and globally relevant research.

## Authors contributions

Conceptualisation: PG, DF, HA, RB, ER, PM

Data curation: PG, DF, HA

Formal analysis: PG

Investigation: PG, HA, DF

Methodology: PG, DF, HA, RB, ER, PM

Supervision: RB, ER, PM

Validation: PG, HA

Visualisation: PG

Writing - original draft: PG

Writing – revisions: PG

Writing - review and editing: PG, DF, HA, RB, ER, PM

## Declaration of Generative AI and AI-assisted technologies in the writing process

During the preparation of this work the authors used Grammarly in order to improve readability and language. After using this tool, the authors reviewed and edited the content as needed and take full responsibility for the content of the publication.

## Funding

This scoping review is independent research and there are no relevant funding sources to declare.

## Declaration of interest

The authors declare that they have no conflict of interest.
