# Supplementary material for: Platform trials in anaesthesia and perioperative medicine: a scoping review
Source: BJA Open. 2026 Jun 2;18:100569. doi: 10.1016/j.bjao.2026.100569 (PMC13251709; doi:10.1016/j.bjao.2026.100569)
Supplement: multimedia component 1 [file mmc1.pdf]

Figure S1: Preferred Reporting Items for Systematic reviews and Meta-Analyses extension for Scoping Reviews (PRISMA-ScR) Checklist

SECTION	ITEM	PRISMA-ScR CHECKLIST ITEM	REPORTED ON PAGE #
**TITLE**			
Title	1	Identify the report as a scoping review.	1
**ABSTRACT**			
Structured summary	2	Provide a structured summary that includes (as applicable): background, objectives, eligibility criteria, sources of evidence, charting methods, results, and conclusions that relate to the review questions and objectives.	2-3
**INTRODUCTION**			
Rationale	3	Describe the rationale for the review in the context of what is already known. Explain why the review questions/objectives lend themselves to a scoping review approach.	4-5
Objectives	4	Provide an explicit statement of the questions and objectives being addressed with reference to their key elements (e.g., population or participants, concepts, and context) or other relevant key elements used to conceptualize the review questions and/or objectives.	4-5
**METHODS**			
Protocol and registration	5	Indicate whether a review protocol exists; state if and where it can be accessed (e.g., a Web address); and if available, provide registration information, including the registration number.	6
Eligibility criteria	6	Specify characteristics of the sources of evidence used as eligibility criteria (e.g., years considered, language, and publication status), and provide a rationale.	7-8
Information sources*	7	Describe all information sources in the search (e.g., databases with dates of coverage and contact with authors to identify additional sources), as well as the date the most recent search was executed.	6-7
Search	8	Present the full electronic search strategy for at least 1 database, including any limits used, such that it could be repeated.	Supp material
Selection of sources of evidence†	9	State the process for selecting sources of evidence (i.e., screening and eligibility) included in the scoping review.	8-9
Data charting process‡	10	Describe the methods of charting data from the included sources of evidence (e.g., calibrated forms or forms that have been tested by the team before their use, and whether data charting was done independently or in duplicate) and any processes for obtaining and confirming data from investigators.	9-10, Supp material
Data items	11	List and define all variables for which data were sought and any assumptions and simplifications made.	Supp material
Critical appraisal of individual sources of evidence§	12	If done, provide a rationale for conducting a critical appraisal of included sources of evidence; describe the methods used and how this information was used in any data synthesis (if appropriate).	N/A
Synthesis of results	13	Describe the methods of handling and summarizing the data that were charted.	10

SECTION	ITEM	PRISMA-ScR CHECKLIST ITEM	REPORTED ON PAGE #
**RESULTS**			
Selection of sources of evidence	14	Give numbers of sources of evidence screened, assessed for eligibility, and included in the review, with reasons for exclusions at each stage, ideally using a flow diagram.	Figure 1
Characteristics of sources of evidence	15	For each source of evidence, present characteristics for which data were charted and provide the citations.	Table 1-2
Critical appraisal within sources of evidence	16	If done, present data on critical appraisal of included sources of evidence (see item 12).	N/A
Results of individual sources of evidence	17	For each included source of evidence, present the relevant data that were charted that relate to the review questions and objectives.	11-13
Synthesis of results	18	Summarize and/or present the charting results as they relate to the review questions and objectives.	Table 1-2
**DISCUSSION**			
Summary of evidence	19	Summarize the main results (including an overview of concepts, themes, and types of evidence available), link to the review questions and objectives, and consider the relevance to key groups.	14-20
Limitations	20	Discuss the limitations of the scoping review process.	19-20
Conclusions	21	Provide a general interpretation of the results with respect to the review questions and objectives, as well as potential implications and/or next steps.	21
**FUNDING**			
Funding	22	Describe sources of funding for the included sources of evidence, as well as sources of funding for the scoping review. Describe the role of the funders of the scoping review.	26

JBI = Joanna Briggs Institute; PRISMA-ScR = Preferred Reporting Items for Systematic reviews and Meta-Analyses extension for Scoping Reviews.
* Where *sources of evidence* (see second footnote) are compiled from, such as bibliographic databases, social media platforms, and Web sites.
† A more inclusive/heterogeneous term used to account for the different types of evidence or data sources (e.g., quantitative and/or qualitative research, expert opinion, and policy documents) that may be eligible in a scoping review as opposed to only studies. This is not to be confused with *information sources* (see first footnote).
‡ The frameworks by Arksey and O'Malley (6) and Levac and colleagues (7) and the JBI guidance (4, 5) refer to the process of data extraction in a scoping review as data charting.
§ The process of systematically examining research evidence to assess its validity, results, and relevance before using it to inform a decision. This term is used for items 12 and 19 instead of "risk of bias" (which is more applicable to systematic reviews of interventions) to include and acknowledge the various sources of evidence that may be used in a scoping review (e.g., quantitative and/or qualitative research, expert opinion, and policy document).

From: Tricco AC, Lillie E, Zarin W, O'Brien KK, Colquhoun H, Levac D, et al. PRISMA Extension for Scoping Reviews (PRISMA-ScR): Checklist and Explanation. Ann Intern Med. ;169:467–473. doi: 10.7326/M18-0850

Figure S2: Database, registry and grey literature search strategy

1. Databases

Medline

1 adaptive clinical trial/ 180

2 Adaptive Clinical Trials as Topic/ 551

3 (Adapt* adj1 (platform* or multiplatform or multi-platform or Bayesian* or frequentist or flexible or trial* or design* or study or studies or clinical or randomi* or control* or phase* or sequential or approach* or method* or model* or test* or framework* or seamless or single-blind or pragmatic or enrichment or dose-finding or hierarch* or multi-stage* or multistage* or continu* reassessment or treatment switch*)).ti,ab,kw,kf. 26568

4 ((Platform* or multiplatform or multi-platform) adj1 (Bayesian* or frequentist or flexible or trial* or design* or study or studies or clinical or randomi* or control* or phase* or sequential or approach* or method* or model* or test* or framework* or seamless or single-blind or pragmatic or enrichment or dose-finding or hierarch* or multi-stage* or multistage* or continu* reassessment or treatment switch*)).ti,ab,kw,kf. 8472

5 (Bayesian* adj1 (frequentist or flexible or trial* or design* or study or studies or clinical or randomi* or control* or phase* or sequential or approach* or method* or model* or test* or framework* or seamless or single-blind or pragmatic or enrichment or dose-finding or hierarch* or multi-stage* or multistage* or continu* reassessment or treatment switch*)).ti,ab,kw,kf. 26154

6 (Frequentist adj1 (flexible or trial* or design* or study or studies or clinical or randomi* or control* or phase* or sequential or approach* or method* or model* or test* or framework* or seamless or single-blind or pragmatic or enrichment or dose-finding or hierarch* or multi-stage* or multistage* or continu* reassessment or treatment switch*)).ti,ab,kw,kf. 1265

7 (Flexible adj1 (trial* or design* or study or studies or clinical or randomi* or control* or phase* or sequential or approach* or method* or model* or test* or framework* or seamless or single-blind or pragmatic or enrichment or dose-finding or hierarch* or multi-stage* or multistage* or continu* reassessment or treatment switch*)).ti,ab,kw,kf. 7884

8 ((umbrella trial* or umbrella design*) and (adapt* or flexible)).ti,ab,kw,kf. 32

9 ((basket trial* or basket design*) and (adapt* or flexible)).ti,ab,kw,kf. 60

10 master protocol*.ti,ab,kw,kf. 334

11 ((drop* or pick* or play* or choose*) adj2 (loser* or winner*)).ti,ab,kw,kf. 185

12 (multi-arm or multiarm or arm-switch*).ti,ab,kw,kf. 1362

13 group sequential.ti,ab,kw,kf. 997

14 or/1-13 70565

15 Perioperative Medicine/ 177

16 exp Perioperative Period/ 106991

17 exp Perioperative Care/ 162644

18 (preoperative or pre-operative or perioperative or peri-operative or intraoperative or intra-operative or postoperative or post-operative).ti,ab,kw,kf. 1076915

19 or/15-18 1180586

20 Surgical Procedures, Operative/ 57441

21 (Surger* or surgic*).ti,ab,kw,kf. 2463549

22 or/20-21 2474410

23 exp "Anesthesia and Analgesia"/ 259950

24 Anesthesiology/ 34097

25 exp Anesthetics/ 271561

26 Anesthesia Department, Hospital/ 1114

27 exp Anesthetists/ 5728

28 an?esthe*.ti,ab,kw,kf. 453936

29 or/23-28 700905

30 exp Intensive Care Units/ 114715

31 exp Critical Care/ 69424

32 Critical Illness/ 42344

33 (intensive care or ICU or NICU or PICU or coronary care unit or respiratory care unit or critical care or critical illness or intensive treatment or intensivist).ti,ab,kw,kf. 305822

34 or/30-33 364596

35 or/19,22,29,34 3681169

36 14 and 35 3905

Embase

1 adaptive clinical trial/ 518

2 "adaptive clinical trial (topic)"/ 139

3 (Adapt* adj1 (platform* or multiplatform or multi-platform or Bayesian* or frequentist or flexible or trial* or design* or study or studies or clinical or randomi* or control* or phase* or sequential or approach* or method* or model* or test* or framework* or seamless or single-blind or pragmatic or enrichment or dose-finding or hierarch* or multi-stage* or multistage* or continu* reassessment or treatment switch*)).ti,ab,kw,kf. 34582

4 ((Platform* or multiplatform or multi-platform) adj1 (Bayesian* or frequentist or flexible or trial* or design* or study or studies or clinical or randomi* or control* or phase* or sequential or approach* or method* or model* or test* or framework* or seamless or single-blind or pragmatic or enrichment or dose-finding or hierarch* or multi-stage* or multistage* or continu* reassessment or treatment switch*)).ti,ab,kw,kf. 14382

5 (Bayesian* adj1 (frequentist or flexible or trial* or design* or study or studies or clinical or randomi* or control* or phase* or sequential or approach* or method* or model* or test* or framework* or seamless or single-blind or pragmatic or enrichment or dose-finding or hierarch* or multi-stage* or multistage* or continu* reassessment or treatment switch*)).ti,ab,kw,kf. 29458

6 (Frequentist adj1 (flexible or trial* or design* or study or studies or clinical or randomi* or control* or phase* or sequential or approach* or method* or model* or test* or framework* or seamless or single-blind or pragmatic or enrichment or dose-finding or hierarch* or multi-stage* or multistage* or continu* reassessment or treatment switch*)).ti,ab,kw,kf. 1491

7 (Flexible adj1 (trial* or design* or study or studies or clinical or randomi* or control* or phase* or sequential or approach* or method* or model* or test* or framework* or seamless or single-blind or pragmatic or enrichment or dose-finding or hierarch* or multi-stage* or multistage* or continu* reassessment or treatment switch*)).ti,ab,kw,kf. 8837

8 ((umbrella trial* or umbrella design*) and (adapt* or flexible)).ti,ab,kw,kf. 66

9 ((basket trial* or basket design*) and (adapt* or flexible)).ti,ab,kw,kf. 125

10 master protocol*.ti,ab,kw,kf. 586

11 ((drop* or pick* or play* or choose*) adj2 (loser* or winner*)).ti,ab,kw,kf. 320

12 (multi-arm or multiarm or arm-switch*).ti,ab,kw,kf. 2006

13 group sequential.ti,ab,kw,kf. 1327

14 or/1-13 89462

15 perioperative medicine/ 956

16 exp perioperative period/ 1312626

17 exp perioperative nursing/ 8145

18 exp perioperative complication/ 1105855

19 (preoperative or pre-operative or perioperative or peri-operative or intraoperative or intra-operative or postoperative or post-operative).ti,ab,kw,kf. 1545630

20 or/15-19 2766556

21 exp surgery/ 6964992

22 (Surger* or surgic*).ti,ab,kw,kf. 3501102

23 or/21-22 7803956

24 exp anesthesiological procedure/ 1133211

25 Anesthesiology/ 26565

26 exp anesthetic agent/ 786119

27 anesthesiologist/ 14498

28 an?esthe*.ti,ab,kw,kf. 659426

29 or/24-28 1958989

30 exp intensive care unit/ 346024

31 intensive care medicine/ 2041

32 critical illness/ 38181

33 intensivist/ 6600

34 (intensive care or ICU or NICU or PICU or coronary care unit or respiratory care unit or critical care or critical illness or intensive treatment or intensivist).ti,ab,kw,kf. 500671

35 or/30-34 617641

36 or/20,23,29,35 9501518

37 14 and 36 11446

Scopus

((TITLE-ABS-KEY ("group sequential")) OR (TITLE-ABS-KEY (multi-arm OR multiarm OR arm-switch*)) OR (TITLE-ABS-KEY ("drop* the loser*" OR "drop* the winner*" OR "pick* the winner*" OR "pick* the loser*" OR "play* the winner*" OR "play* the loser*" OR "Choose* the winner*" OR "choose* the loser*")) OR (TITLE-ABS-KEY ("master protocol*")) OR (TITLE-ABS-KEY (("basket trial*" OR "basket design*") AND (adapt* OR flexible))) OR (TITLE-ABS-KEY (("umbrella trial*" OR "umbrella design*") AND (adapt* OR flexible))) OR (TITLE-ABS-KEY (flexible W/0 (seamless OR single-blind OR pragmatic OR enrichment OR dose-finding OR hierarch* OR multi-stage* OR multistage* OR "continu* reassessment" OR "treatment switch*"))) OR (TITLE-ABS-KEY (flexible W/0 (trial* OR design* OR study OR studies OR clinical OR randomi* OR control* OR phase* OR sequential OR approach* OR method* OR model* OR test* OR framework*))) OR (TITLE-ABS-KEY (frequentist W/0 (seamless OR single-blind OR pragmatic OR enrichment OR dose-finding OR hierarch* OR multi-stage* OR multistage* OR "continu* reassessment" OR "treatment switch*"))) OR (TITLE-ABS-KEY (frequentist W/0 (flexible OR trial* OR design* OR study OR studies OR clinical OR randomi* OR control* OR phase* OR sequential OR approach* OR method* OR model* OR test* OR framework*))) OR (TITLE-ABS-KEY (bayesian* W/0 (seamless OR single-blind OR pragmatic OR enrichment OR dose-finding OR hierarch* OR multi-stage* OR multistage* OR "continu* reassessment" OR "treatment switch*"))) OR (TITLE-ABS-KEY (bayesian* W/0 (frequentist OR flexible OR trial* OR design* OR study OR studies OR clinical OR randomi* OR control* OR phase* OR sequential OR approach* OR method* OR model* OR test* OR framework*))) OR (TITLE-ABS-KEY ((platform* OR multiplatform OR multi-platform) W/0 (test* OR framework* OR seamless OR single-blind OR pragmatic OR enrichment OR dose-finding OR hierarch* OR multi-stage* OR multistage* OR "continu* reassessment" OR "treatment switch*"))) OR (TITLE-ABS-KEY ((platform* OR multiplatform OR multi-platform) W/0 (bayesian* OR frequentist OR flexible OR trial* OR design* OR study OR studies OR clinical OR randomi* OR control* OR phase* OR sequential OR approach* OR method* OR model*))) OR (TITLE-ABS-KEY (adapt* W/0 (method* OR model* OR test* OR framework* OR seamless OR single-blind OR pragmatic OR enrichment OR dose-finding OR hierarch* OR multi-stage* OR multistage* OR "continu* reassessment" OR "treatment switch*"))) OR (TITLE-ABS-KEY (adapt* W/0 (platform* OR multiplatform OR multi-platform OR bayesian* OR frequentist OR flexible OR trial* OR design* OR study OR studies OR clinical OR randomi* OR control* OR phase* OR sequential OR approach*)))) AND ((TITLE-ABS-KEY (preoperative OR pre-

operative OR perioperative OR peri-operative OR intraoperative OR intra-operative OR postoperative OR post-operative OR surger* OR surgic* OR anesthe* OR anaesthe*)) OR (TITLE-ABS-KEY ("intensive care" OR icu OR nicu OR picu OR "coronary care unit" OR "respiratory care unit" OR "critical care" OR "critical illness" OR "intensive treatment" OR intensivist)))

Expanded search lines to show how the above search was constructed

1. TITLE-ABS-KEY (adapt* W/0 (platform* OR multiplatform OR multi-platform OR bayesian* OR frequentist OR flexible OR trial* OR design* OR study OR studies OR clinical OR randomi* OR control* OR phase* OR sequential OR approach*)

2. TITLE-ABS-KEY (adapt* W/0 (method* OR model* OR test* OR framework* OR seamless OR single-blind OR pragmatic OR enrichment OR dose-finding OR hierarch* OR multi-stage* OR multistage* OR "continu* reassessment" OR "treatment switch*"))

3. TITLE-ABS-KEY ((platform* OR multiplatform OR multi-platform) W/0 (bayesian* OR frequentist OR flexible OR trial* OR design* OR study OR studies OR clinical OR randomi* OR control* OR phase* OR sequential OR approach* OR method* OR model*))

4. TITLE-ABS-KEY ((platform* OR multiplatform OR multi-platform) W/0 (test* OR framework* OR seamless OR single-blind OR pragmatic OR enrichment OR dose-finding OR hierarch* OR multi-stage* OR multistage* OR "continu* reassessment" OR "treatment switch*"))

5. TITLE-ABS-KEY (bayesian* W/0 (frequentist OR flexible OR trial* OR design* OR study OR studies OR clinical OR randomi* OR control* OR phase* OR sequential OR approach* OR method* OR model* OR test* OR framework*))

6. TITLE-ABS-KEY (bayesian* W/0 (seamless OR single-blind OR pragmatic OR enrichment OR dose-finding OR hierarch* OR multi-stage* OR multistage* OR "continu* reassessment" OR "treatment switch*"))

7. TITLE-ABS-KEY (frequentist W/0 (flexible OR trial* OR design* OR study OR studies OR clinical OR randomi* OR control* OR phase* OR sequential OR approach* OR method* OR model* OR test* OR framework*))

8. TITLE-ABS-KEY (frequentist W/0 (seamless OR single-blind OR pragmatic OR enrichment OR dose-finding OR hierarch* OR multi-stage* OR multistage* OR "continu* reassessment" OR "treatment switch*"))

9. TITLE-ABS-KEY (flexible W/0 (trial* OR design* OR study OR studies OR clinical OR rando

mi* OR control* OR phase* OR sequential OR approach* OR method* OR model* OR test* OR framework*))

10. TITLE-ABS-KEY (flexible W/0 (seamless OR single-blind OR pragmatic OR enrichment OR dose-finding OR hierarch* OR multi-stage* OR multistage* OR "continu* reassessment" OR "treatment switch*"))
11. TITLE-ABS-KEY (("umbrella trial*" OR "umbrella design*") AND (adapt* OR flexible))
12. TITLE-ABS-KEY (("basket trial*" OR "basket design*") AND (adapt* OR flexible))
13. TITLE-ABS-KEY ("master protocol*")
14. TITLE-ABS-KEY ("drop* the loser*" OR "drop* the winner*" OR "pick* the winner*" OR "pick* the loser*" OR "play* the winner*" OR "play* the loser*" OR "Choose* the winner*" OR "choose* the loser*")
15. TITLE-ABS-KEY (multi-arm OR multiarm OR arm-switch*)
16. TITLE-ABS-KEY ("group sequential")
17. (TITLE-ABS-KEY ("group sequential")) OR (TITLE-ABS-KEY (multi-arm OR multiarm OR arm-switch*)) OR (TITLE-ABS-KEY ("drop* the loser*" OR "drop* the winner*" OR "pick* the winner*" OR "pick* the loser*" OR "play* the winner*" OR "play* the loser*" OR "Choose* the winner*" OR "choose* the loser*")) OR (TITLE-ABS-KEY ("master protocol*")) OR (TITLE-ABS-KEY (("basket trial*" OR "basket design*") AND (adapt* OR flexible))) OR (TITLE-ABS-KEY (("umbrella trial*" OR "umbrella design*") AND (adapt* OR flexible))) OR (TITLE-ABS-KEY (flexible W/0 (seamless OR single-blind OR pragmatic OR enrichment OR dose-finding OR hierarch* OR multi-stage* OR multistage* OR "continu* reassessment" OR "treatment switch*"))) OR (TITLE-ABS-KEY (flexible W/0 (trial* OR design* OR study OR studies OR clinical OR randomi* OR control* OR phase* OR sequential OR approach* OR method* OR model* OR test* OR framework*))) OR (TITLE-ABS-KEY (frequentist W/0 (seamless OR single-blind OR pragmatic OR enrichment OR dose-finding OR hierarch* OR multi-stage* OR multistage* OR "continu* reassessment" OR "treatment switch*"))) OR (TITLE-ABS-KEY (frequentist W/0 (flexible OR trial* OR design* OR study OR studies OR clinical OR randomi* OR control* OR phase* OR sequential OR approach* OR method* OR model* OR test* OR framework*))) OR (TITLE-ABS-KEY (bayesian* W/0 (seamless OR single-blind OR pragmatic OR enrichment OR dose-finding OR hierarch* OR multi-

stage* OR multistage* OR "continu* reassessment" OR "treatment switch*"))) OR (TITLE-ABS-KEY (bayesian* W/0 (frequentist OR flexible OR trial* OR design* OR study OR studies OR clinical OR randomi* OR control* OR phase* OR sequential OR approach* OR method* OR model* OR test* OR framework*))) OR (TITLE-ABS-KEY ((platform* OR multiplatform OR multi-platform) W/0 (test* OR framework* OR seamless OR single-blind OR pragmatic OR enrichment OR dose-finding OR hierarch* OR multi-stage* OR multistage* OR "continu* reassessment" OR "treatment switch*"))) OR (TITLE-ABS-KEY ((platform* OR multiplatform OR multi-platform) W/0 (bayesian* OR frequentist OR flexible OR trial* OR design* OR study OR studies OR clinical OR randomi* OR control* OR phase* OR sequential OR approach* OR method* OR model*))) OR (TITLE-ABS-KEY (adapt* W/0 (method* OR model* OR test* OR framework* OR seamless OR single-blind OR pragmatic OR enrichment OR dose-finding OR hierarch* OR multi-stage* OR multistage* OR "continu* reassessment" OR "treatment switch*"))) OR (TITLE-ABS-KEY (adapt* W/0 (platform* OR multiplatform OR multi-platform OR bayesian* OR frequentist OR flexible OR trial* OR design* OR study OR studies OR clinical OR randomi* OR control* OR phase* OR sequential OR approach*)))

18. TITLE-ABS-KEY (preoperative OR pre-operative OR perioperative OR peri-operative OR intraoperative OR intra-operative OR postoperative OR post-operative OR surger* OR surgic* OR anesthe* OR anaesthe*)

19. TITLE-ABS-KEY ("intensive care" OR icu OR nicu OR picu OR "coronary care unit" OR "respiratory care unit" OR "critical care" OR "critical illness" OR "intensive treatment" OR intensivist)

20. (TITLE-ABS-KEY (preoperative OR pre-operative OR perioperative OR peri-operative OR intraoperative OR intra-operative OR postoperative OR post-operative OR surger* OR surgic* OR anesthe* OR anaesthe*)) OR (TITLE-ABS-KEY ("intensive care" OR icu OR nicu OR picu OR "coronary care unit" OR "respiratory care unit" OR "critical care" OR "critical illness" OR "intensive treatment" OR intensivist))

21. ((TITLE-ABS-KEY ("group sequential")) OR (TITLE-ABS-KEY (multi-arm OR multiarm OR arm-switch*)) OR (TITLE-ABS-KEY ("drop* the loser*" OR "drop* the winner*" OR "pick* the winner*" OR "pick* the loser*" OR "play* the winner*" OR "play* the loser*" OR "Choose* the winner*" OR "choose* the loser*")) OR (TITLE-ABS-KEY ("master protocol*")) OR (TITLE-ABS-KEY (("basket trial*" OR "basket design*") AND (adapt* OR flexible))) OR (TITLE-ABS-KEY (("umbrella trial*" OR "umbrella design*") AND (adapt* OR flexible))) OR (TITLE-ABS-KEY (flexible W/0 (seamless OR single-

blind OR pragmatic OR enrichment OR dose-finding OR hierarch* OR multi-stage* OR multistage* OR "continu* reassessment" OR "treatment switch*"))) OR (TITLE-ABS-KEY (flexible W/0 (trial* OR design* OR study OR studies OR clinical OR randomi* OR control* OR phase* OR sequential OR approach* OR method* OR model* OR test* OR framework*))) OR (TITLE-ABS-KEY (frequentist W/0 (seamless OR single-blind OR pragmatic OR enrichment OR dose-finding OR hierarch* OR multi-stage* OR multistage* OR "continu* reassessment" OR "treatment switch*"))) OR (TITLE-ABS-KEY (frequentist W/0 (flexible OR trial* OR design* OR study OR studies OR clinical OR randomi* OR control* OR phase* OR sequential OR approach* OR method* OR model* OR test* OR framework*))) OR (TITLE-ABS-KEY (bayesian* W/0 (seamless OR single-blind OR pragmatic OR enrichment OR dose-finding OR hierarch* OR multi-stage* OR multistage* OR "continu* reassessment" OR "treatment switch*"))) OR (TITLE-ABS-KEY (bayesian* W/0 (frequentist OR flexible OR trial* OR design* OR study OR studies OR clinical OR randomi* OR control* OR phase* OR sequential OR approach* OR method* OR model* OR test* OR framework*))) OR (TITLE-ABS-KEY ((platform* OR multiplatform OR multi-platform) W/0 (test* OR framework* OR seamless OR single-blind OR pragmatic OR enrichment OR dose-finding OR hierarch* OR multi-stage* OR multistage* OR "continu* reassessment" OR "treatment switch*"))) OR (TITLE-ABS-KEY ((platform* OR multiplatform OR multi-platform) W/0 (bayesian* OR frequentist OR flexible OR trial* OR design* OR study OR studies OR clinical OR randomi* OR control* OR phase* OR sequential OR approach* OR method* OR model*))) OR (TITLE-ABS-KEY (adapt* W/0 (method* OR model* OR test* OR framework* OR seamless OR single-blind OR pragmatic OR enrichment OR dose-finding OR hierarch* OR multi-stage* OR multistage* OR "continu* reassessment" OR "treatment switch*"))) OR (TITLE-ABS-KEY (adapt* W/0 (platform* OR multiplatform OR multi-platform OR bayesian* OR frequentist OR flexible OR trial* OR design* OR study OR studies OR clinical OR randomi* OR control* OR phase* OR sequential OR approach*)))) AND ((TITLE-ABS-KEY (preoperative OR pre-operative OR perioperative OR peri-operative OR intraoperative OR intra-operative OR postoperative OR post-operative OR surger* OR surgic* OR anesthe* OR anaesthe*)) OR (TITLE-ABS-KEY ("intensive care" OR icu OR nicu OR picu OR "coronary care unit" OR "respiratory care unit" OR "critical care" OR "critical illness" OR "intensive treatment" OR intensivist)))

CINAHL Complete

S59	S33 AND S58	1,587
S58	S40 OR S44 OR S51 OR S57	1,267,120
S57	S52 OR S53 OR S54 OR S55 OR S56	172,412
S56	AB ("intensive care" OR icu OR nicu OR picu OR "coronary care unit" OR "respiratory care unit" OR "critical care" OR "critical illness" OR "intensive treatment" OR intensivist)	100,100
S55	TI ("intensive care" OR icu OR nicu OR picu OR "coronary care unit" OR "respiratory care unit" OR "critical care" OR "critical illness" OR "intensive treatment" OR intensivist)	63,360
S54	(MH "Critical Illness")	15,806
S53	(MH "Critical Care+")	34,061
S52	(MH "Intensive Care Units+")	78,864
S51	S45 OR S46 OR S47 OR S48 OR S49 OR S50	155,451
S50	AB an#esthe*	60,799
S49	TI an#esthe*	40,702
S48	(MH "Anesthesiologists")	1,832
S47	(MH "Anesthetics+")	49,237
S46	(MH "Anesthesiology")	6,757
S45	(MH "Anesthesia and Analgesia+")	82,019
S44	S41 OR S42 OR S43	1,003,562
S43	AB (surger* OR surgic*)	373,299
S42	TI (surger* OR surgic*)	184,879
S41	(MH "Surgery, Operative+")	776,767
S40	S34 OR S35 OR S36 OR S37 OR S38 OR S39	261,816
S39	AB (preoperative OR pre-operative OR perioperative OR peri-operative OR intraoperative OR intra-operative OR postoperative OR post-operative)	176,426
S38	TI (preoperative OR pre-operative OR perioperative OR peri-operative OR intraoperative OR intra-operative OR postoperative OR post-operative)	73,001
S37	(MH "Perioperative Nursing+")	17,509
S36	(MH "Perioperative Medicine")	51

S35	(MH "Preoperative Period+")	8,819
S34	(MH "Perioperative Care+")	67,028
S33	S1 OR S2 OR S3 OR S4 OR S5 OR S6 OR S7 OR S8 OR S9 OR S10 OR S11 OR S12 OR S13 OR S14 OR S15 OR S16 OR S17 OR S18 OR S19 OR S20 OR S21 OR S22 OR S23 OR S24 OR S25 OR S26 OR S27 OR S28 OR S29 OR S30 OR S31 OR S32	13,493
S32	AB "group sequential"	171
S31	TI "group sequential"	97
S30	AB (multi-arm OR multiarm OR arm-switch*)	256
S29	TI (multi-arm OR multiarm OR arm-switch*)	193
S28	AB ("drop* the loser*" OR "drop* the winner*" OR "pick* the winner*" OR "pick* the loser*" OR "play* the winner*" OR "play* the loser*" OR "Choose* the winner*" OR "choose* the loser*")	27
S27	TI ("drop* the loser*" OR "drop* the winner*" OR "pick* the winner*" OR "pick* the loser*" OR "play* the winner*" OR "play* the loser*" OR "Choose* the winner*" OR "choose* the loser*")	27
S26	AB "master protocol*"	89
S25	TI "master protocol*"	40
S24	AB (("basket trial*" OR "basket design*") AND (adapt* OR flexible))	11
S23	TI (("basket trial*" OR "basket design*") AND (adapt* OR flexible))	2
S22	AB (("umbrella trial*" OR "umbrella design*") AND (adapt* OR flexible))	7
S21	TI (("umbrella trial*" OR "umbrella design*") AND (adapt* OR flexible))	1
S20	AB (flexible N0 (seamless OR single-blind OR pragmatic OR enrichment OR dose-finding OR hierarch* OR multi-stage* OR multistage* OR "continu* reassessment" OR "treatment switch*")	11
S19	TI (flexible N0 (seamless OR single-blind OR pragmatic OR enrichment OR dose-finding OR hierarch* OR multi-stage* OR multistage* OR "continu* reassessment" OR "treatment switch*")	2

S18	AB (flexible N0 (trial* OR design* OR study OR studies OR clinical OR randomi* OR control* OR phase* OR sequential OR approach* OR method* OR model* OR test* OR framework*)	1,335
S17	TI (flexible N0 (trial* OR design* OR study OR studies OR clinical OR randomi* OR control* OR phase* OR sequential OR approach* OR method* OR model* OR test* OR framework*)	159
S16	AB (frequentist N0 (seamless OR single-blind OR pragmatic OR enrichment OR dose-finding OR hierarch* OR multi-stage* OR multistage* OR "continu* reassessment" OR "treatment switch*"))	1
S15	TI (frequentist N0 (seamless OR single-blind OR pragmatic OR enrichment OR dose-finding OR hierarch* OR multi-stage* OR multistage* OR "continu* reassessment" OR "treatment switch*"))	0
S14	AB (frequentist N0 (flexible OR trial* OR design* OR study OR studies OR clinical OR randomi* OR control* OR phase* OR sequential OR approach* OR method* OR model* OR test* OR framework*))	291
S13	TI (frequentist N0 (flexible OR trial* OR design* OR study OR studies OR clinical OR randomi* OR control* OR phase* OR sequential OR approach* OR method* OR model* OR test* OR framework*))	14
S12	AB (bayesian* N0 (seamless OR single-blind OR pragmatic OR enrichment OR dose-finding OR hierarch* OR multi-stage* OR multistage* OR "continu* reassessment" OR "treatment switch*"))	768
S11	TI (bayesian* N0 (seamless OR single-blind OR pragmatic OR enrichment OR dose-finding OR hierarch* OR multi-stage* OR multistage* OR "continu* reassessment" OR "treatment switch*"))	183
S10	AB ((bayesian* N0 (frequentist OR flexible OR trial* OR design* OR study OR studies OR clinical OR randomi* OR control* OR phase* OR sequential OR approach* OR method* OR model* OR test* OR framework*))	2,496
S9	TI ((bayesian* N0 (frequentist OR flexible OR trial* OR design* OR study OR studies OR clinical OR randomi* OR control* OR phase* OR sequential OR approach* OR method* OR model* OR test* OR framework*))	764
S8	AB ((platform* OR multiplatform OR multi-platform) N0 (test* OR framework* OR seamless OR single-blind OR pragmatic OR enrichment OR dose-finding OR hierarch* OR	256

	multi-stage* OR multistage* OR "continu* reassessment" OR "treatment switch*"))	
S7	TI ((platform* OR multiplatform OR multi-platform) N0 (test* OR framework* OR seamless OR single-blind OR pragmatic OR enrichment OR dose-finding OR hierarch* OR multi-stage* OR multistage* OR "continu* reassessment" OR "treatment switch*"))	50
S6	AB ((platform* OR multiplatform OR multi-platform) N0 (bayesian* OR frequentist OR flexible OR trial* OR design* OR study OR studies OR clinical OR randomi* OR control* OR phase* OR sequential OR approach* OR method* OR model*))	853
S5	TI ((platform* OR multiplatform OR multi-platform) N0 (bayesian* OR frequentist OR flexible OR trial* OR design* OR study OR studies OR clinical OR randomi* OR control* OR phase* OR sequential OR approach* OR method* OR model*))	265
S4	AB (adapt* N0 (method* OR model* OR test* OR framework* OR seamless OR single-blind OR pragmatic OR enrichment OR dose-finding OR hierarch* OR multi-stage* OR multistage* OR "continu* reassessment" OR "treatment switch*"))	3,372
S3	TI (adapt* N0 (method* OR model* OR test* OR framework* OR seamless OR single-blind OR pragmatic OR enrichment OR dose-finding OR hierarch* OR multi-stage* OR multistage* OR "continu* reassessment" OR "treatment switch*"))	926
S2	AB ((adapt* N0 ("platform*" OR "multiplatform" OR "multi-platform" OR "Bayesian*" OR "frequentist" OR "flexible" OR "trial*" OR "design*" OR "study" OR "studies" OR "clinical" OR "randomi*" OR "control*" OR "phase*" OR "sequential" OR "approach*")))	2,481
S1	TI (adapt* N0 ("platform*" OR "multiplatform" OR "multi-platform" OR "Bayesian*" OR "frequentist" OR "flexible" OR "trial*" OR "design*" OR "study" OR "studies" OR "clinical" OR "randomi*" OR "control*" OR "phase*" OR "sequential" OR "approach*"))	886

Web of Science Core Collection, All Editions

Search: TS=(preoperative or pre-operative or perioperative or peri-operative or intraoperative or intra-operative or postoperative or post-operative or surger* or surgic* or an$esthe* or "intensive care" or ICU or NICU or PICU or "coronary care unit" or "respiratory care unit" or "critical care" or "critical illness" or "intensive treatment" or intensivist) Results: 3325308

Search: #1 OR #2 OR #3 OR #4 OR #5 OR #6 OR #7 OR #8 OR #9 OR #10 OR #11 Results: 281650

Search: TS=(adapt* NEAR/0 (platform* or multiplatform or multi-platform or Bayesian* or frequentist or flexible or trial* or design* or study or studies or clinical or randomi* or control* or phase* or sequential or approach* or method* or model* or test* or framework* or seamless or single-blind or pragmatic or enrichment or dose-finding or hierarch* or multi-stage* or multistage* or "continu* reassessment" or "treatment switch*")) Results: 143627

Search: TS=((Platform* or multiplatform or multi-platform) NEAR/0 (Bayesian* or frequentist or flexible or trial* or design* or study or studies or clinical or randomi* or control* or phase* or sequential or approach* or method* or model* or test* or framework* or seamless or single-blind or pragmatic or enrichment or dose-finding or hierarch* or multi-stage* or multistage* or "continu* reassessment" or "treatment switch*")) Results: 29861

Search: TS=(Bayesian* NEAR/0 (frequentist or flexible or trial* or design* or study or studies or clinical or randomi* or control* or phase* or sequential or approach* or method* or model* or test* or framework* or seamless or single-blind or pragmatic or enrichment or dose-finding or hierarch* or multi-stage* or multistage* or "continu* reassessment" or "treatment switch*")) Results: 73484

Search: TS=(Frequentist NEAR/0 (flexible or trial* or design* or study or studies or clinical or randomi* or control* or phase* or sequential or approach* or method* or model* or test* or framework* or seamless or single-blind or pragmatic or enrichment or dose-finding or hierarch* or multi-stage* or multistage* or "continu* reassessment" or "treatment switch*")) Results: 2318

Search: TS=(Flexible NEAR/0 (trial* or design* or study or studies or clinical or randomi* or control* or phase* or sequential or approach* or method* or model* or test* or framework* or seamless or single-blind or pragmatic or enrichment or dose-finding or hierarch* or multi-stage* or multistage* or "continu* reassessment" or "treatment switch*")) Results: 28490

Search: TS=(("umbrella trial*" or "umbrella design*") and (adapt* or flexible)) Results: 40

Search: TS=(("basket trial*" or "basket design*") and (adapt* or flexible)) Results: 89

Search: TS=("master protocol*") Results: 430

Search: TS=((drop* or pick* or play* or choose*) NEAR/1 (loser* or winner*)) Results: 699

Search: TS=(multi-arm or multiarm or arm-switch*) Results: 6673

Search: TS=("group sequential") Results: 1517

Cochrane Library (Wiley)

#1 MeSH descriptor: [Adaptive Clinical Trial] explode all trees 0

#2 MeSH descriptor: [Adaptive Clinical Trials as Topic] explode all trees 29

#3 adapt* NEAR/0 (platform* or multiplatform or multi-platform or Bayesian* or frequentist or flexible or trial* or design* or study or studies or clinical or randomi* or control* or phase* or sequential or approach* or method* or model* or test* or framework* or seamless or single-blind or pragmatic or enrichment or dose-finding or hierarch* or multi-stage* or multistage* or (continual NEXT reassess*) or (treatment NEXT switch*)):ti,ab,kw 109

#4 (Platform* or multiplatform or multi-platform) NEAR/0 (Bayesian* or frequentist or flexible or trial* or design* or study or studies or clinical or randomi* or control* or phase* or sequential or approach* or method* or model* or test* or framework* or seamless or single-blind or pragmatic or enrichment or dose-finding or hierarch* or multi-stage* or multistage* or (continual NEXT reassess*) or (treatment NEXT switch*)):ti,ab,kw 43

#5 Bayesian* NEAR/0 (frequentist or flexible or trial* or design* or study or studies or clinical or randomi* or control* or phase* or sequential or approach* or method* or model* or test* or framework* or seamless or single-blind or pragmatic or enrichment or dose-finding or hierarch* or multi-stage* or multistage* or (continual NEXT reassess*) or (treatment NEXT switch*)):ti,ab,kw 10

#6 Frequentist NEAR/0 (flexible or trial* or design* or study or studies or clinical or randomi* or control* or phase* or sequential or approach* or method* or model* or test* or framework* or seamless or single-blind or pragmatic or enrichment or dose-finding or hierarch* or multi-stage* or multistage* or (continual NEXT reassess*) or (treatment NEXT switch*)):ti,ab,kw 0

#7 Flexible NEAR/0 (trial* or design* or study or studies or clinical or randomi* or control* or phase* or sequential or approach* or method* or model* or test* or framework* or seamless or single-blind or pragmatic or enrichment or dose-finding or hierarch* or multi-stage* or multistage* or (continual NEXT reassess*) or (treatment NEXT switch*)):ti,ab,kw 0

#8 ((umbrella NEXT trial*) or (umbrella NEXT design*)) and (adapt* or flexible):ti,ab,kw 18

#9 ((basket NEXT trial*) or (basket NEXT design*)) and (adapt* or flexible):ti,ab,kw 22

#10 master NEXT protocol*:ti,ab,kw 257

#11 (drop* or pick* or play* or choose*) NEAR/1 (loser* or winner*):ti,ab,kw 77

#12 multi-arm or multiarm or arm-switch*:ti,ab,kw 1739

#13 "group sequential":ti,ab,kw 450

#14 {OR #1-#13} 2690

#15 MeSH descriptor: [Perioperative Care] explode all trees 14931

#16 MeSH descriptor: [Perioperative Period] explode all trees 11271

#17 MeSH descriptor: [Perioperative Medicine] explode all trees 1

#18 preoperative or pre-operative or perioperative or peri-operative or intraoperative or intra-operative or postoperative or post-operative:ti,ab,kw 210667

#19 {OR #15-#18} 212920

#20 MeSH descriptor: [Surgical Procedures, Operative] explode all trees 171621

#21 Surger* or surgic*:ti,ab,kw 372737

#22 {OR #20-#21} 427749

#23 MeSH descriptor: [Anesthesia and Analgesia] explode all trees 34947

#24 MeSH descriptor: [Anesthesiology] explode all trees 588

#25 MeSH descriptor: [Anesthetics] explode all trees 19314

#26 MeSH descriptor: [Anesthesia Department, Hospital] explode all trees 6

#27 MeSH descriptor: [Anesthetists] explode all trees 191

#28 an*esthe*:ti,ab,kw 134503

#29 {OR #23-#28} 141408

#30 MeSH descriptor: [Intensive Care Units] explode all trees 6114

#31 MeSH descriptor: [Critical Care] explode all trees 3029

#32 MeSH descriptor: [Critical Illness] explode all trees 3727

#33 "intensive care" or ICU or NICU or PICU or "coronary care unit" or "respiratory care unit" or "critical care" or "critical illness" or "intensive treatment" or intensivist:ti,ab,kw 74421

#34 {OR #30-#33} 74609

#35 #19 OR #22 OR #29 OR #34 543555

#36 #14 AND #35893

2. Registries

Clinical trials.gov

"Adaptive platform" OR "Adaptive platform trial" OR "Basket design" OR "Basket factorial MAMS" OR "Basket trial" OR "Drop-the-loser" OR "Factorial-MAMS" OR "MAMS" OR "MAMS basket" OR "MAMS factorial" OR "MAMS-platform" OR "Master protocol" OR "Multi-arm multi-stage" OR "Multi-arm multi-stage platform trial" OR "Multi-arm platform" OR "Multi platform" OR "Multi-stage platform" OR "Multiplatform" OR "Platform clinical trial" OR "Platform design" OR "Platform protocol" OR "Platform study" OR "Platform trial" OR "Response adaptive randomisation" OR "Umbrella design" OR "Umbrella trial" AND ("Anesthesiology" OR "Anesthesia and Analgesia" OR "Anesthetics" OR "Perioperative care" OR "Peri-operative care" OR "Perioperative Medicine" OR "Peri-operative medicine" OR "Perioperative period" OR "Peri-operative period" OR "Surgery" OR "Surgical" OR "Surgical Procedures" OR “intensive care” OR “ICU” OR “NICU” OR “PICU” OR “coronary care unit” OR “respiratory care unit” OR “critical care” OR “critical illness” OR “intensive treatment”)

National Institute for Health and Care Research (NIHR)

"Adaptive platform" | "Adaptive platform trial" | "Basket design" | "Basket factorial MAMS" | "Basket trial" | "Drop-the-loser" | "Factorial-MAMS" | "MAMS" | "MAMS basket" | "MAMS factorial" | "MAMS-platform" | "Master protocol" | "Multi-arm multi-stage" | "Multi-arm multi-stage platform trial" | "Multi-arm platform" | "Multi platform" | "Multi-stage platform" | "Multiplatform" | "Platform clinical trial" | "Platform design" | "Platform protocol" | "Platform study" | "Platform trial" | "Response adaptive randomisation" | "Umbrella design" | "Umbrella trial" + ("Anesthesiology" | "Anesthesia and Analgesia" | "Anesthetics" | "Perioperative care" | "Peri-operative care" | "Perioperative Medicine" | "Peri-operative medicine" | "Perioperative period" | "Peri-operative period" | "Surgery" | "Surgical" | "Surgical Procedures" | “intensive care” | “ICU” | “NICU” | “PICU” | “coronary care unit” | “respiratory care unit” | “critical care” | “critical illness” | “intensive treatment”)

International Standardised Randomised Control Trial Number Registry (ISRCTN)

"Adaptive platform" OR "Adaptive platform trial" OR "Basket design" OR "Basket factorial MAMS" OR "Basket trial" OR "Drop-the-loser" OR "Factorial-MAMS" OR "MAMS" OR "MAMS basket" OR "MAMS factorial" OR "MAMS-platform" OR "Master protocol" OR "Multi-arm multi-stage" OR "Multi-arm multi-stage platform trial" OR "Multi-arm platform" OR "Multi platform" OR "Multi-stage platform" OR "Multiplatform" OR "Platform clinical trial" OR "Platform design" OR "Platform protocol" OR "Platform study" OR "Platform trial" OR "Response adaptive randomisation" OR "Umbrella design" OR "Umbrella trial" AND ("Anesthesiology" OR "Anesthesia and Analgesia" OR "Anesthetics" OR "Perioperative care" OR "Peri-operative care" OR "Perioperative Medicine" OR "Peri-operative medicine" OR "Perioperative period" OR "Peri-operative period" OR "Surgery" OR "Surgical" OR "Surgical Procedures"

OR “intensive care” OR “ICU” OR “NICU” OR “PICU” OR “coronary care unit” OR “respiratory care unit” OR “critical care” OR “critical illness” OR “intensive treatment”)

Australian New Zealand Clinical Trials Registry (ANZCTR)

“Adaptive Platform” OR “Adaptive platform trial” OR “Platform trial perioperative medicine” OR “platform trial ICU” OR “platform trial critical care”

World Health Organization International Clinical Trials Registry Platform (ICTRP)

"Adaptive platform" OR "Adaptive platform trial" OR "Basket design" OR "Basket factorial MAMS" OR "Basket trial" OR "Drop-the-loser" OR "Factorial-MAMS" OR "MAMS" OR "MAMS basket" OR "MAMS factorial" OR "MAMS-platform" OR "Master protocol" OR "Multi-arm multi-stage" OR "Multi-arm multi-stage platform trial" OR "Multi-arm platform" OR "Multi platform" OR "Multi-stage platform" OR "Multiplatform" OR "Platform clinical trial" OR "Platform design" OR "Platform protocol" OR "Platform study" OR "Platform trial" OR "Response adaptive randomisation" OR "Umbrella design" OR "Umbrella trial" AND ("Anesthesiology" OR "Anesthesia and Analgesia" OR "Anesthetics" OR "Perioperative care" OR "Peri-operative care" OR "Perioperative Medicine" OR "Peri-operative medicine" OR "Perioperative period" OR "Peri-operative period" OR "Surgery" OR "Surgical" OR "Surgical Procedures" OR “intensive care” OR “ICU” OR “NICU” OR “PICU” OR “coronary care unit” OR “respiratory care unit” OR “critical care” OR “critical illness” OR “intensive treatment”)

3. Other sources

Europe PMC

"Adaptive platform" OR "Adaptive platform trial" OR "Basket design" OR "Basket factorial MAMS" OR "Basket trial" OR "Drop-the-loser" OR "Factorial-MAMS" OR "MAMS" OR "MAMS basket" OR "MAMS factorial" OR "MAMS-platform" OR "Master protocol" OR "Multi-arm multi-stage" OR "Multi-arm multi-stage platform trial" OR "Multi-arm platform" OR "Multi platform" OR "Multi-stage platform" OR "Multiplatform" OR "Platform clinical trial" OR "Platform design" OR "Platform protocol" OR "Platform study" OR "Platform trial" OR "Response adaptive randomisation" OR "Umbrella design" OR "Umbrella trial" AND ("Anesthesiology" OR "Anesthesia and Analgesia" OR "Anesthetics" OR "Perioperative care" OR "Peri-operative care" OR "Perioperative Medicine" OR "Peri-operative medicine" OR "Perioperative period" OR "Peri-operative period" OR "Surgery" OR "Surgical" OR "Surgical Procedures" OR “intensive care” OR “ICU” OR “NICU” OR “PICU” OR “coronary care unit” OR “respiratory care unit” OR “critical care” OR “critical illness” OR “intensive treatment”)

medRxiv

“Adaptive Platform” OR “Adaptive platform trial” OR “Platform trial perioperative medicine” OR “platform trial ICU” OR “platform trial critical care”

Pubmed Central (preprint)

"Adaptive platform" OR "Adaptive platform trial" OR "Basket design" OR "Basket factorial MAMS" OR "Basket trial" OR "Drop-the-loser" OR "Factorial-MAMS" OR "MAMS" OR "MAMS basket" OR "MAMS factorial" OR "MAMS-platform" OR "Master protocol" OR "Multi-arm multi-stage" OR "Multi-arm multi-stage platform trial" OR "Multi-arm platform" OR "Multi platform" OR "Multi-stage platform" OR "Multiplatform" OR "Platform clinical trial" OR "Platform design" OR "Platform protocol" OR "Platform study" OR "Platform trial" OR "Response adaptive randomisation" OR "Umbrella design" OR "Umbrella trial" AND ("Anesthesiology" OR "Anesthesia and Analgesia" OR "Anesthetics" OR "Perioperative care" OR "Peri-operative care" OR "Perioperative Medicine" OR "Peri-operative medicine" OR "Perioperative period" OR "Peri-operative period" OR "Surgery" OR "Surgical" OR "Surgical Procedures" OR “intensive care” OR “ICU” OR “NICU” OR “PICU” OR “coronary care unit” OR “respiratory care unit” OR “critical care” OR “critical illness” OR “intensive treatment”)

Open Science Framework (OSF)

“Adaptive Platform” OR “Adaptive platform trial” OR “Platform trial perioperative medicine” OR “platform trial ICU” OR “platform trial critical care”

Zenodo

"Adaptive platform" OR "Adaptive platform trial" OR "Basket design" OR "Basket factorial MAMS" OR "Basket trial" OR "Drop-the-loser" OR "Factorial-MAMS" OR "MAMS" OR "MAMS basket" OR "MAMS factorial" OR "MAMS-platform" OR "Master protocol" OR "Multi-arm multi-stage" OR "Multi-arm multi-stage platform trial" OR "Multi-arm platform" OR "Multi platform" OR "Multi-stage platform" OR "Multiplatform" OR "Platform clinical trial" OR "Platform design" OR "Platform protocol" OR "Platform study" OR "Platform trial" OR "Response adaptive randomisation" OR "Umbrella design" OR "Umbrella trial" AND ("Anesthesiology" OR "Anesthesia and Analgesia" OR "Anesthetics" OR "Perioperative care" OR "Peri-operative care" OR "Perioperative Medicine" OR "Peri-operative medicine" OR "Perioperative period" OR "Peri-operative period" OR "Surgery" OR "Surgical" OR "Surgical Procedures" OR “intensive care” OR “ICU” OR “NICU” OR “PICU” OR “coronary care unit” OR “respiratory care unit” OR “critical care” OR “critical illness” OR “intensive treatment”)

Table S1: Decision framework template for identifying platform trials in anaesthesia and peri-operative medicine. * A study was included only if all criteria were met. **Domain relates to an area within which several interventions are compared e.g. drug class or treatment class. †Peri-operative medicine was defined as care delivered within a defined surgical episode, spanning the pre-operative, intra-operative, and postoperative phases, and involving anaesthesia within a coordinated multidisciplinary pathway.[25]

**Criteria**	**Yes/ No**	**Decision**
Trial included multiple interventions within one or more domains		
Master or core protocol		
Add new questions to trial by adding new treatments and/or subgroups		
Anesthesia and/or peri-operative medicine population		

Table S2: Data extraction template

	Field	Details/ Notes
**General**	Information source	Manuscript (database search), website (grey literature)
	Master Protocol Title	
	Registry & ID	ClinicalTrials.gov, ISRCTN, ANZCTR, EUCTR, etc.
	Registry ID	
	Contact / Lead Institution	Name, email, institution
	Duration	Estimated Start/ End date
	Trial Title/ Official	Full trial title
	Trial Status (as per ClinicalTrials.gov)	Recruiting, Active, Completed, Terminated, Suspended
**Population & Setting**	Aim	
	Patient Population	Age, sex, comorbidities, ASA class
	Surgical Type	Cardiac, abdominal, orthopaedic, obstetric, mixed, etc.
	Anaesthetic Context	General anaesthesia, regional, sedation, ICU, mixed
	Geographic Setting	Country, multicentre/national/international
**Trial Design**	Domains	Single vs multiple
	Design	Multi-arm, multifactorial, REMAP-style, master protocol
	Randomization Type	Response-adaptive, Bayesian, frequentist, stratified
	Control Type	Concurrent, non-concurrent, usual care
	Adaptive Features	Arm addition/removal, interim analyses, stopping rules
	Blinding	Open-label, single-blind, double-blind
	Statistical Models	
**Interventions / Domains**	Feasibility trial conducted	
	Intervention Name / Domain	Drug, technique, bundle, or management strategy
	Comparator	Standard care, other interventions
	Dose / Timing / Delivery	Details of intervention administration
	Were interventions compared to the control or each other as primary analysis	

**Outcomes**	Number of participating arms	Allocation ratios between the intervention and control group
	Strata/ Sub groups	
	Primary Outcomes	Clinical or patient-centered, composite outcomes
**Statistical & Analytical Approaches**	Secondary Outcomes	Safety, process, or surrogate outcomes
	Sample Size / Adaptation	Adaptive sample size, re-estimation, power considerations
	Statistical Models	Hierarchical, Bayesian, frequentist, mixed-effects
	Non-concurrent Control Methods	Borrowing approaches, adjustment methods
	Multiplicity Handling	Correction or adaptive design strategy
	Interim evaluations	
	Prespecified interim analysis	
	Trial adjustments made at interim analysis	
	Goal of interim analyses	
	Statistical simulations	
	Method of randomisation	Simple, RAR, ratio, minimisation, other
	Statistical adjustment for temporal drift	Yes/ No
	Statistical software	
**Governance & Operations**	Statistical rules merits/ rules were used to guide adaptations	Criteria for arm dropping, graduating to next stage or introducing a new intervention, when new interventions added – who made the decision
	Oversight Committees	DSMB, Steering Committee, Scientific Advisory Board
	Ethical / Regulatory Approvals	National/institutional ethics
	Data Infrastructure	Central database, eCRF, multi-site integration
	Sponsor	Industry, academic, government
**Implementation & Feasibility**	Funding source	
	Recruitment Strategy	Inclusion/exclusion, enrolment rate
**Reporting & Transparency**	Challenges / Barriers	Operational, regulatory, logistical

	Protocol Availability	Published / online / supplementary
	Publications / Preprints	Links or references
**Gaps / Notes**	Reporting Standards	CONSORT, SPIRIT, platform trial-specific
	Research Gaps	Methodological, population, intervention gaps
	Observations	Notes on trends, innovations, unusual features

Table S3: Full titles of platform trials in anaesthesia and peri-operative medicine. UPMC, University of Pittsburgh Medical Center; SSI, surgical site infection.

Acronym	Platform trial title
UPMC REMAP[26]	Randomised, Embedded, Multifactorial, Adaptive Platform Trial for Optimising Surgical Outcomes at UPMC
MARLIN[27]	A Stratified, Multi-arm, multi-site Randomised Platform Trial Aiming to Reduce the incidence of Post-operative SSI
PROMPT[28]	Perioperative Medicine Platform Trial
PROTECT-Surg[29]	Preventing Pulmonary Complications in Surgical Patients at Risk of COVID-19
REMAP-Periop[30]	Randomised, Embedded, Multifactorial Adaptive Platform for Perioperative Medicine at UPMC (UPMC REMAP): Core Protocol - Enhanced Recovery Protocols (ERP)
ROADMAP[31]	Randomised Arthroplasty infection worldwide Multidomain Adaptive Platform trial
ROSSINI-2[32]	Reduction of Surgical Site Infection Using Several Novel Interventions
ROSSINI-Platform[33]	Reduction Of Surgical Site Infection with a platform trial utilising a 'Basket-MAMS' design
PROTECT[34]	A national perioperative platform trial to improve outcomes for surgical patients

Table S4: Income level classification of countries conducting platform trials in anaesthesia and peri-operative medicine. * Trials are categorised according to the income level of participating countries using the World Bank country income classification at the time of data extraction. HIC, high-income country; LMIC, lower-middle-income country; UMIC, upper-middle-income country; UK, United Kingdom; USA, United States of America.

**Trial (acronym)**	**Trial site/s**	**Countries involved**	**Income-level classification**
UPMC REMAP[26]	Multicentre-national	USA	HIC
MARLIN[27]	Multicentre-international	Benin; Rwanda; Ghana; India; Mexico; Nigeria; South Africa	LMIC
PROMPT[28]	Multicentre-international	Australia; New Zealand	HIC
PROTECT-Surg[29]	Multicentre-international	Nigeria; Rwanda	LMIC
REMAP-Periop[30]	Multicentre-national	USA	HIC
ROADMAP[31]	Multicentre-international	Australia; New Zealand; Canada; UK	HIC
ROSSINI-2[32]	Multicentre-national	UK	HIC
ROSSINI-Platform[33]	Multicentre-national	UK	HIC
PROTECT[34]	Multicentre-national	UK	HIC

Table S5: Operational characteristics of included platform trials – sponsorship models, funding and collaborators.

**Platform trial (acronym)**	**Sponsor type**	**Name of sponsor**	**Collaborators**	**Sources of funding**
UPMC REMAP[26]	Academic	University of Pittsburgh Medical Center, United States of America	Berry Consultants, USA	University of Pittsburgh Medical Center Immune Transplant and Therapy Center grant (IPA2019#8)
MARLIN[27]	Academic	University of Birmingham, United Kingdom	Kigali University Teaching Hospital, Rwanda<br><br>Université d'Abomey-Calavi, Benin	Not reported
PROMPT[28]	Academic	Monash University, Australia	The Alfred, Victoria, Australia	Victorian Medical Research Acceleration Fund grant (ID GA-F4668961-1110)<br><br>Health Research Council grant, New Zealand
PROTECT-Surg[29]	Academic	University of Birmingham, United Kingdom	Christian Medical College and Hospital, Ludhiana, India<br><br>Ministry of Health, Ghana<br>University of Lagos, Nigeria<br><br>Kigali University Teaching Hospital, Rwanda<br><br>Hospital Español Veracruz, Mexico<br>Université d'Abomey-Calavi, Benin	Not reported

			University of Witwatersrand, South Africa<br><br>University of Edinburgh, UK<br><br>Istituto Clinico Humanitas, Italy<br><br>University of Cape Town, South Africa	
REMAP-Periop[30]	Academic	University of Pittsburgh Medical Center, United States of America	Berry Consultants, USA	Beckwith Institute of the University of Pittsburgh Medical Center grant (BECKW15880)
ROADMAP[31]	Academic	University of Newcastle, Australia	Hunter Medical Research Institute, Australia<br><br>The University of Western Australia, Australia<br><br>Michael Garron Hospital, Canada<br><br>Aotearoa Clinical Trials, New Zealand<br><br>Exeter Orthopaedic Trial unit, UK<br><br>University of Sydney, Australia<br><br>University of Melbourne, Australia<br><br>The Peter Doherty Institute for Infection and Immunity, Australia	National Health and Medical Research Council, Australia (Grant # 2024015)<br><br>Health Research Council, New Zealand (Grant # 24/395)<br><br>Canadian Institutes of Health Research, Canada (Grant 2025-03-05 # 540279)<br><br>Ministere de la Sante et de la Prevention, France (ReCH MIE 2024 # ECTZ 342126)

ROSSINI 2[32]	Academic	University of Birmingham, United Kingdom	National Institute for Health Research, UK<br><br>University College London, UK	The National Institute for Health and Care Research - 'Health Technology Assessment' programme - Grant reference number: NIHR156728<br><br>Extension award (project ID NIHR160509)
ROSSINI-Platform[33]	Academic	University of Birmingham, United Kingdom	Not reported	National Institute for Health and Care Research - 'Health Technology Assessment' programme - Grant reference number: NIHR156728<br><br>(£10 million funding over 5 years)
PROTECT[34]	Academic	Queen Mary University of London, United Kingdom	Not reported	Barts Charity, UK (G-002514)<br><br>Academy of Medical Science, UK (SGL029\1104)<br><br>British Journal of Anaesthesia (WKR0-2023-0016)

Table S6: Operational characteristics of included platform trials – data infrastructure, consent models and governance structures. CRF, case report form; CTU, clinical trials unit; EHR, electronic health record; EMR, electronic medical record; REC, research ethics committee

**Platform trial (acronym)**	**Data capture and storage**	**Database**	**Consent model**	**Oversight committees**
UPMC REMAP[26]	Learning health system embedded - EHR screening, enrolment integrated into clinic visits, outcomes derived from EHR data	Embedded platform	Prospective informed consent (EHR-integrated)	Strategic oversight, independent data monitoring, statistical monitoring
MARLIN[27]	Multi-site integration	Not reported	Not reported	Independent data monitoring
PROMPT[28]	Not reported	Not reported	Not reported	Strategic oversight, independent data monitoring, advisory support
PROTECT-Surg[29]	Not reported	Not reported	Consent model varied by site under local ethics approvals	Central coordination (CTU), operational management, oversight and monitoring
REMAP-Periop[30]	Institutional software interfacing with EMR for recruitment, enrolment, allocation and data collection	Embedded platform	Waiver of consent	Strategic oversight, independent data monitoring, statistical monitoring
ROADMAP[31]	Standardised data collection via web-based electronic CRF portal	Web-based electronic CRF (central database)	Governed locally by site-specific ethics approvals	Global and regional strategic oversight, domain-specific working groups

ROSSINI 2[32]	Electronic CRFs and paper CRFs (transcribed to electronic CRFs by ROSSINI 2 trials office)	REDCap database hosted at University of Birmingham	Prospective informed consent under REC approval at each site	Operational management, strategic oversight, independent monitoring, central coordination (CTU)
ROSSINI-Platform[33]	Not reported	Not reported	Not reported	Central management, strategic oversight, independent monitoring
PROTECT[34]	Electronic capture in central PROTECT database with paper CRFs transcribed locally into database	Central PROTECT database – hosted within Trusted Research Environment at Swansea University	Ethics-approved consent (London - South East Research Ethics Committee)	Multi-level governance, strategic oversight, operational management, comparison-level oversight

[*]Governance structures have been grouped into functional categories: strategic oversight (e.g. TSC, PSC, CSC), independent monitoring (e.g. DSMB, DMEC, DMC), statistical monitoring (e.g. SMC), operational management (e.g. TMG, PMG, CMG), and central coordination (e.g. clinical trials units such as BCTU or MRC CTU). BCTU, Birmingham clinical trials unit; CMG, clinical management group; CSC, clinical steering committee; DMC, data monitoring committee; DMEC, data monitoring and ethics board; DSMB, data and safety monitoring board; MRC CTU, Medical research council clinical trials unit; PMG, project management group; PSC, programme steering committee; SMC, statistical monitoring committee; TMG, trial management group; TSC, trial steering committee
